# Country governance of antimicrobial resistance (AMR) surveillance: observations on global progress and aid programme effectiveness using data from the Tracking AMR Country Self-Assessment Survey (TrACSS)

**DOI:** 10.1186/s12992-025-01179-4

**Published:** 2026-01-07

**Authors:** Archie Drake, Isabel Sassoon, Jennifer Armitage, Syed Abbas, Rebecca Maudling, Ankur Gupta-Wright, Alan Serrano, Anila Shafa, Tim Shorten

**Affiliations:** 1 Independent Consultant, Dar es Salaam, United Republic of Tanzania; 2 Brunel University of London, London, UK; 3 LAMP Development Organisation Ltd, Edinburgh, UK; 4 Institute of Development Studies, Brighton, UK; 5 Independent Consultant, London, UK; 6 Imperial College London, London, UK; 7 Itad Ltd, Brighton, UK

**Keywords:** Antimicrobial resistance (AMR), Public health surveillance, One health, Governance, Policy, Regulation, Evaluation, Development effectiveness, TrACSS

## Abstract

**Background:**

AMR is an important global public health challenge, but there is a lack of good data on response progress. This study addresses country governance of antimicrobial resistance (AMR) surveillance, considering changes in responses to the Tracking Antimicrobial Resistance Country Self-Assessment Survey (TrACSS) between 2019 and 2024. Its first objective is to describe progress under the global action agenda on AMR. Its second objective is to assess the effectiveness of a major development aid intervention to encourage action against AMR, the United Kingdom (UK)-funded Fleming Fund (FF). The study applies a pragmatic approach to analysis, involving descriptive exploration and difference-in-differences methodology.

**Results:**

Governance of AMR surveillance in low- and middle-income countries generally strengthened over the five years to 2024, converging with high-income countries (HIC). South-East Asian countries reported relatively strong gains, a striking exception to limited global progress. Globally, over fifteen indicators, clear progress was only reported in four: two on strengthening underlying AMR surveillance systems, in both human health (HH) and animal health (AH); and two on regulatory frameworks in AH. FF-supported countries reported strengthening HH surveillance systems more than comparable countries, even accounting for income-group differences. The standardised change score for the ‘national surveillance system for AMR in humans’ was 0.11 higher in FF-supported than in other Official Development Assistance (ODA)-eligible countries (95% confidence level, *p* = 0.046). FF-supported countries also reported greater progress on other topics, such as use of surveillance data for decision-making in AH. FF-supported countries were approximately a quarter (25%) more likely to respond ‘yes’ on this topic. Additional reflections on the strengths and weaknesses of TrACSS data are discussed in detail.

**Conclusion:**

Our study adds empirical observations about country governance systems as they relate to AMR surveillance efforts. The idea of progressive global momentum, with South-East Asia playing a key role, disrupts established perceptions of European leadership and obstacles to change. From an aid effectiveness perspective, this study indicates that FF strengthened country HH and AH surveillance systems. It also points to institutional changes in how data are used in administrative decision-making processes and, potentially, in the translation of evidence into policy, programmes and regulation.

**Clinical trial number:**

Not applicable.

**Supplementary information:**

The online version contains supplementary material available at 10.1186/s12992-025-01179-4.

## Background

This study focuses on country governance of antimicrobial resistance (AMR) surveillance as a function of two global mechanisms:

1. The global action agenda on AMR, international response to preserve effective treatment and promote disease prevention as currently elaborated under the Global Action Plan (GAP) [1].
2. The Fleming Fund (FF), a major global development aid programme to develop AMR surveillance systems funded by the United Kingdom (UK).

It aims to provide an independent assessment of progress against these two mechanisms, using the Tracking AMR Country Self-Assessment Survey (TrACSS) [2] to inform future thinking and action.

### AMR surveillance and governance at the country level

AMR is a vital international challenge. Estimates show that AMR contributed to the death of nearly 5 million people in 2019, a figure that looks set to rise to more than 8 million in 2050 [3, 4].

Effective action to counter AMR depends on developing improved information about the incidence and trends of drug-resistant infections through **surveillance** [5]. But efforts to strengthen AMR surveillance have proved challenging, especially where relevant systems do not already exist [6].

The complex and interdisciplinary nature of AMR as a public health challenge makes developing AMR surveillance systems difficult [7]:

- In addition to critical laboratory capacity to generate data on susceptibility, AMR response decisions may require information about antimicrobial use and consumption (AMU/C) as well as clinical contexts.
- Surveillance systems are often required to supply information for use across the sectors involved when taking a One Health (OH) approach, including human, animal and environmental health (HH, AH and EH).
- Surveillance is potentially relevant to ‘AMR-sensitive’ as well as ‘AMR-specific’ interventions, in other words to actions serving other agendas but with AMR-relevant implications as well as to actions primarily intended to address AMR [8].

Defining an appropriate strategic focus for AMR surveillance, responding to and coordinating the interests of relevant stakeholders across sectors and disciplines, requires effective **governance**.

### Existing research on AMR country governance

Most existing research on AMR governance at country level focuses on development and implementation of National Action Plans on AMR (NAPs). Anderson et al’s 2019 proposal of a governance framework for the development and assessment of NAPs was foundational to this approach [9]. The framework proposes national action on AMR as a matter of 18 domains featuring in a cycle over three areas: policy design; implementation tools; and monitoring & evaluation. Surveillance is included as one of the eight domains under implementation tools.

There is also some existing work on country governance as a possible explanatory factor for estimates of AMR incidence. Maugeri et al’s work on AMR in European countries applied and built on prior global analysis [10], concluding that two sets of factors appear to be more important drivers of AMR incidence than consumption rates: first, resource constraints like health system infrastructure and health expenditure; and second, wider governance factors like rule of law, political stability and government effectiveness [11].

### Focus of this paper: country governance of AMR surveillance

One existing study proposes governance principles specifically for AMR surveillance. This study defines governance conceptually, for ‘design and evaluation’ purposes, as ‘a set of strategies, rules, norms, principles, and procedures that frame the operation and implementation of surveillance to inform decisions’ [12]. We refer to ‘country governance of AMR surveillance’ pragmatically, for observational purposes, as a set of TrACSS topics which are relevant to surveillance. This includes questions on the technical surveillance system itself, as well as questions which address intended uses of surveillance or resulting actions.

### Objective 1: Assess progress under the global action agenda on AMR

The first objective of this study is to assess progress under the AMR global action agenda in terms of country governance of AMR surveillance. Over the last decade, United Nations (UN) systems^1^ have guided national efforts to develop NAPs on AMR through the GAP. The GAP set out five objectives, one of which was to ‘strengthen the knowledge and evidence base through surveillance and research’.^2^

Current assessments of country governance of AMR tend to emphasise lack of progress. Successive studies applied the Anderson framework using TrACSS and other data available for 2020–21 and 2021–22, highlighting concerns about the extent to which NAPs were being implemented, especially in lower-income countries [13, 14]. For lower- and middle- income countries, resource constraints had emerged as a crucial limitation by that time [15].

Sharper criticisms have suggested a lack of substance to influence on country governance, with NAPs exhibiting isomorphic tendencies; low- and middle-income countries apparently engaging in mimicry and high-income countries (HIC) in posturing [16].

### Objective 2: Assess FF effectiveness as a major development aid initiative on AMR surveillance

The second objective of this paper is to assess the effectiveness of a major aid-funded programme to strengthen AMR surveillance. The UK, along with various other European countries, has sought a lead role in global governance as part of its approach on AMR, *inter alia* through the Fleming Fund [17].

The Fleming Fund (FF) is a flagship UK government programme to counter AMR, committing up to £475 m over the period April 2016 to March 2026. It operates as a surveillance-based programme with a OH approach, engaging with 20+ low- and middle-income countries across Africa and Asia to encourage AMR-related data generation and use. FF has been implemented by the UK Department of Health & Social Care (DHSC) [18]. A 2023 independent evaluation report on FF achievements concluded that progress had been made in developing the foundations for AMR surveillance in partner countries, focusing its recommendations on ways to build towards higher-level outcomes such as data use for policy and sustainable investment [19]. There is a need for evidence about the achievement of higher-level outcomes, which is challenging due to lack of good quality data and evidence on health system outcomes, especially in low- and middle-income countries [20].

### TrACSS as self-assessment data

TrACSS is a global survey designed to monitor NAP implementation [21, 22]. The data are increasingly used in research and evaluation. In research, TrACSS has been mainly used for cross-sectional studies [23], with its potential for considering trends over time only exploited, to the authors’ knowledge, in one study to date [24]. In evaluation, the Organisation for Economic Co-operation and Development (OECD) drew on TrACSS data for its 2023 AMR policy brief; and the World Bank Pandemic Fund plans to use some TrACSS data for annual reporting to the Pandemic Fund Secretariat [25, 26].

## Methods

This study applied a pragmatic approach, addressing its objectives over two successive stages of analysis:

1. Descriptive statistics on global trends (data exploration addressing the first objective of assessing progress under the global action agenda on AMR).
2. Difference-in-differences estimation of apparent change in FF countries relative to comparator groups (a quasi-experimental method for addressing the second objective of assessing FF effectiveness).

Analysis was undertaken using the R software environment [27].

### TrACSS data

Both analysis stages used the same TrACSS data published on the Global Database websites [28, 29].

Two TrACSS rounds were chosen for before-and-after comparison:

- 2019 data (survey responses received in the 3^rd^ TrACSS round, featuring modified questions enabling more consistent matching with later round responses); and
- 2024 data (survey responses received in the 8^th^ TrACSS round, the latest available at the time of undertaking this study).

Variables were selected from the wider range available in TrACSS data to assemble a panel of 15 topics for the study organised into 5 groups, as shown in Table 1. Selection prioritised broad relevance to the FF Theory of Change [30] based on review of TrACSS questions using domain expertise, including scope for possible unintended results.

**Table 1:**
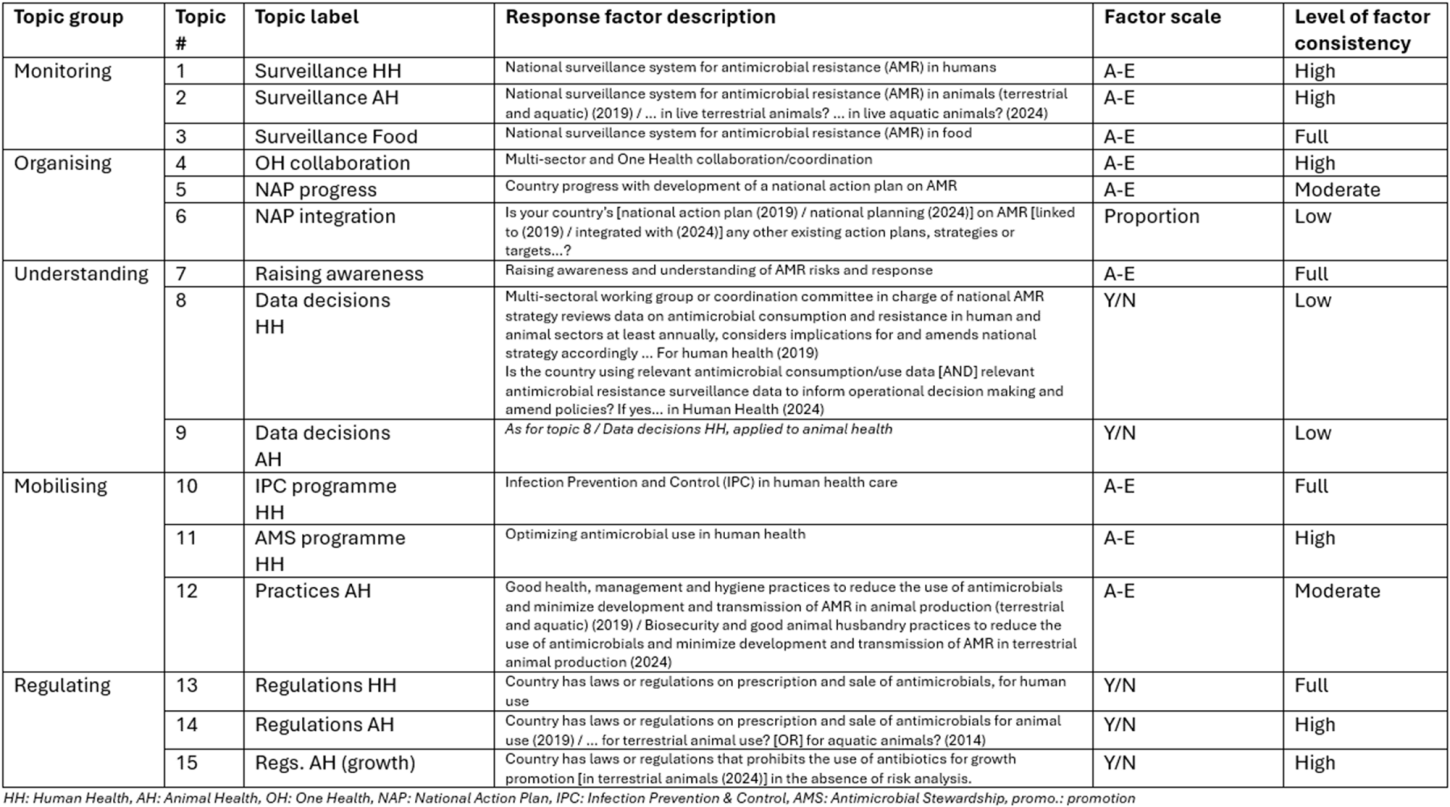
Study topics

TrACSS response variables were assigned numeric values, with higher values for more positive progress in each case. The 2019 value was subtracted from the 2024 value to obtain a numeric change score for each country on each topic. The change scores operated on different scales. Some ranged from −1 to 1 (mainly from yes/no answers and for the proportional approach used for topic 6), whereas others ranged from −4 to 4 (for questions that used the 5-level A-E response scale in TrACSS). All scores were standardised to the −1 to 1 scale for cross-topic comparison.

It is important to note that changes were made to the TrACSS questionnaire between 2019 and 2024 rounds, with variations in factor consistency over time affecting validity of observations about change in study topics in different ways. Table 1 shows factor consistency levels to aid readers to bear this in mind.

See Additional File 1 – Technical Annex – for full details on Theory of Change, variable handling and factor consistency.

### Stage 1: Describing global trends in country governance of AMR surveillance

*A.* Initial exploration adopted a conventional descriptive approach, summarising distributions of country responses across study topics in 2019, 2024 and the resulting change scores. It was only possible to calculate change scores for countries which responded to relevant questions in both 2019 and 2024.

*B.* Global variations were then additionally explored using 2024 country classifications as follows:

- World Bank income classifications [31]; and
- WHO regions (included in TrACSS data).

### Stage 2: Difference-in-differences approach to Fleming Fund (FF) effectiveness

*A.* FF effectiveness was considered using a quasi-experimental design inspired by applications of the difference-in-differences method in impact evaluation [32].

A binary treatment group identifier variable identified 22 countries^3^ which have benefitted from FF interventions, compiled from publicly available data on the FF website at end 2024 [18].

**Fig. 1:**
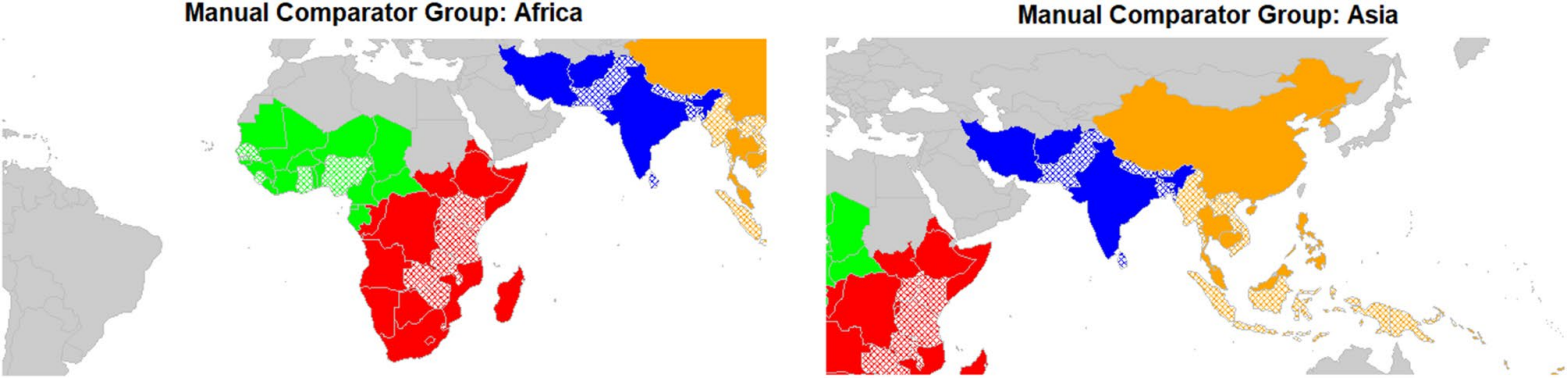
Maps showing ‘manual’ comparator group. Colours show FF region classifications: West Africa (green), East and Southern Africa (red), South Asia (blue) & South-East Asia (orange)

Various control group identifiers were considered as part of the analysis, building on observations from Stage 1. In addition to variables already described, the following factors were added:

- Official Development Assistance (ODA) eligibility as defined by the Organisation for Economic Co-operation and Development (OECD) Development Assistance Committee for 2024 [33].
- A manually identified comparator group based on geographical proximity to FF-supported countries (see Fig. 1).

Analysis focused on describing the apparent difference between treatment and control group change scores for study topics between 2019 and 2024.

*B.* Apparent differences based on FF beneficiary country status were then tested using the Kruskal-Wallis test.

Two further proxy variables were added to outline the nature of FF intervention:

- An estimate of the amount of funding mobilised, using the sum of all Country Grant (CG) budgets for a country specified on the FF website (expressed in £ GBP).
- An estimate of the duration of FF support, measuring time elapsed since the start date of the first CG for a country given on the FF website until June 2024 (expressed in months).

Associations between proxy variables for the nature of FF support and country change scores for study topics were tested using the Spearman’s rank correlation method.

*C.* A final step was to build regression models to test the most significant results on apparent difference between FF and other countries. Country change scores for 2019–2024 were modelled as the dependent variable using binomial regression (positive change scores assigned value ‘1’ and nil or negative change scores ‘0’), with a range of other data alongside FF treatment variables. This approach served to test other potential explanations for apparent differences between FF treatment and control groups (confounding factors in indications of FF effectiveness).

Following the approach adopted in existing TrACSS-based studies of country AMR incidence estimates (see Background), we assembled a further panel of country indicator data:

- A selection of World Development Indicators for 2018 [34], including:

o demographic (population, population density, fertility rate, life expectancy);
o economic (Gross Domestic Product, growth rate, agricultural production indicators);
o governance (corruption, government effectiveness, regulatory quality, rule of law, accountability); and
o health system-related (health expenditure, tuberculosis prevalence).

- AMR burden estimates for 2019 from the Institute for Health Metrics and Evaluation (IHME) generated as part of the Global Research on Antimicrobial Resistance (GRAM) project [35], including both deaths associated with and attributable to AMR.
- Antimicrobial consumption estimates for 2018, also generated under the GRAM project using a spatial modelling approach [36, 37].
- Survey data from a Global Survey of Experts on Antimicrobial Resistance (GSEAR) in low- and middle-income countries undertaken for research purposes in 2021 [38].

Co-correlation matrices were used to select a subset of variables for modelling. Stepwise logistic regression testing was then used to produce a minimum viable model excluding any FF-related variables. Finally, an FF treatment group identifier variable was added to this model to assess its effect size and significance in model context.

## Results

### Stage 1A: Global country change scores 2019–2024

Table 2 below shows distributions of 2019–2024 change scores for study topics across all countries, as well as underlying 2019 and 2024 response counts and means. Most topic change score distributions exhibited a positive mean and a mode value of zero. Overall, this shows limited positive global change on country governance of AMR surveillance over the period.

**Table 2:**
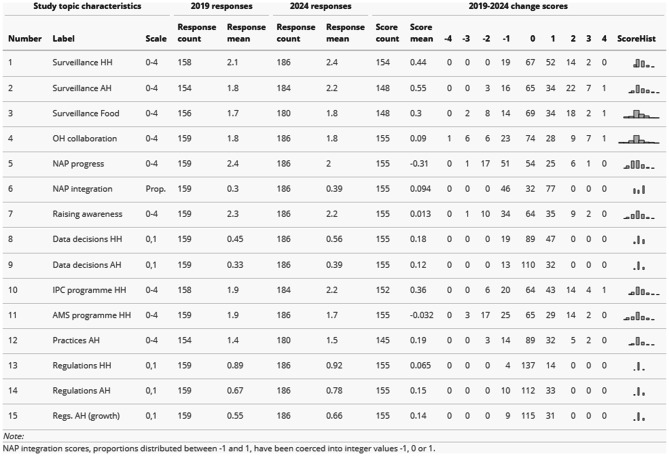
Global change scores

**Fig. 2:**
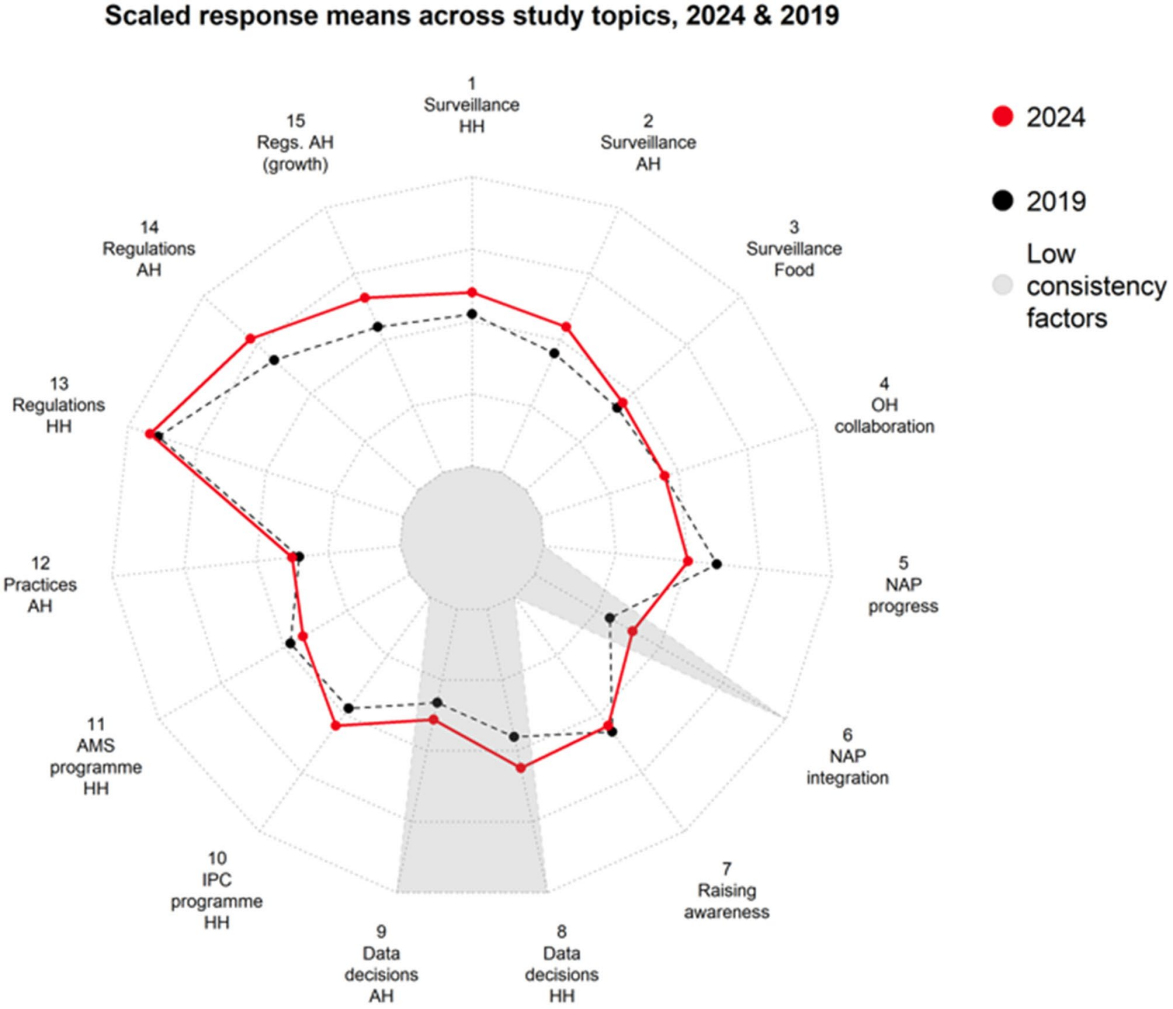
Radar plot comparing 2024 to 2019 country response means

2024 response means are plotted against 2019 response means in Fig. 2 below, with standardised scaling to enable comparison across study topics. This gives a general sense of areas of relative reported strength and weakness. Country responses tended to be more positive for some areas in 2019 than in others. Reading clockwise around the chart, integration of NAPs with other policies (topic 6) and reviewing relevant AMR data for strategic decision-making across HH and AH (topics 8 and 9) appear to have been areas of relative weakness in 2019. Regulatory frameworks in HH (topic 13) were reported to have been relatively common.

Some improvement in the reported strength of national HH and AH AMR surveillance systems (topics 1 and 2) occurred over the period 2019–2024, plus progress towards addressing the apparent weakness in review of AMR data when considering strategy (topics 8 and 9). AH regulation also appears to be a topic on which countries made progress (topics 14 and 15). The topics on which country governance reporting tended to decline slightly were NAP progress (topic 5) and HH AMS (topic 11). At least in the case of NAP progress (topic 5), this may be partly due to factor inconsistencies in the TrACSS indicators, with response grades becoming more challenging in 2024 than they were in 2019 (see Technical Annex).

**Fig. 3:**
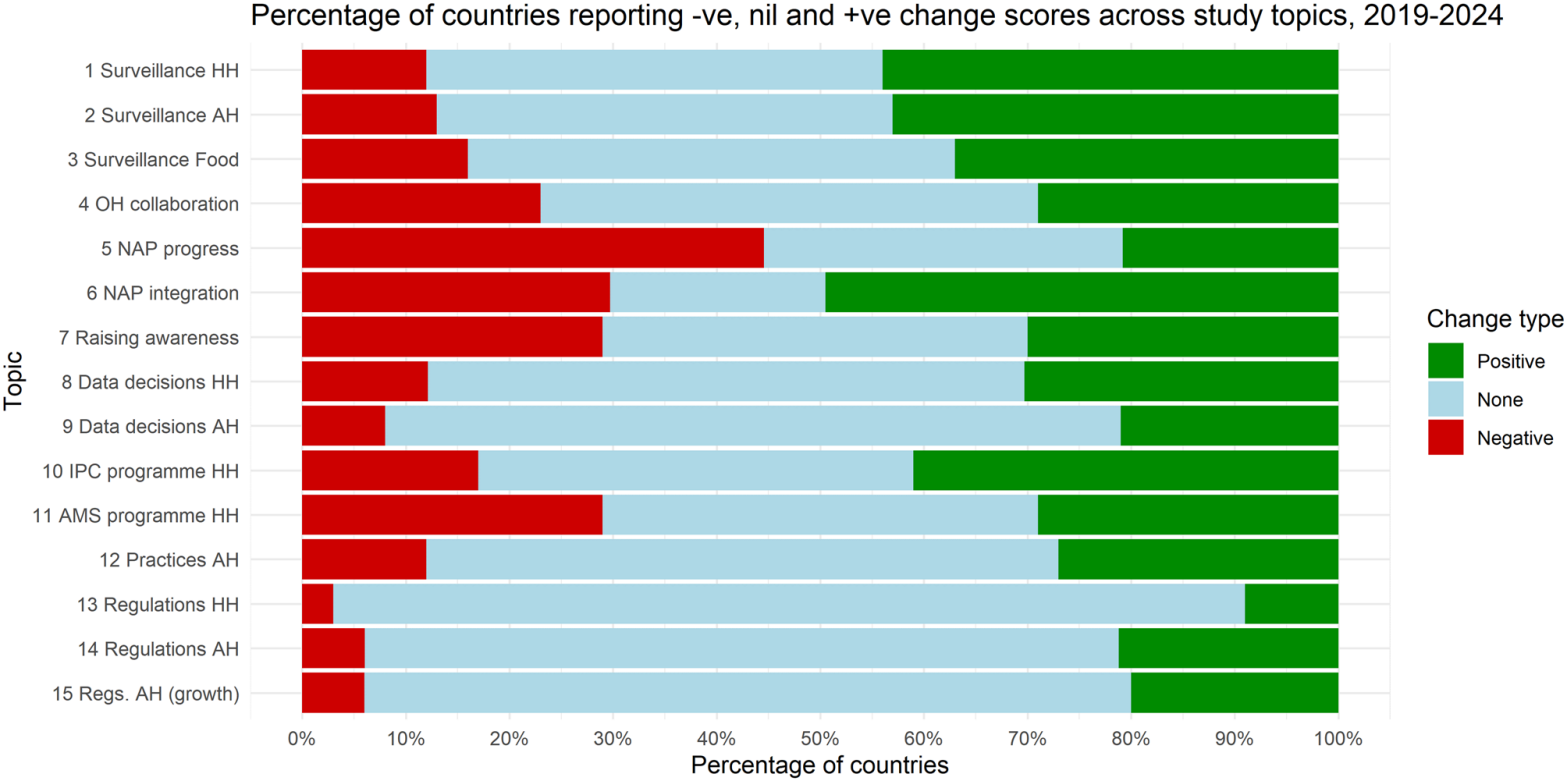
Stacked bar chart showing percentages

Figure 3 represents classification of change scores into simply positive, no change or negative categories, giving a better sense of how differences between 2019 and 2024 responses are distributed across countries. For example, stability in global change scores in the case of OH coordination (topic 4) conceals considerable dynamism in country trajectories, with reporting in about a quarter of countries suggesting progress and in another quarter degeneration over this period.

### Stage 1B: Differences in country change by income level and region

**Fig. 4:**
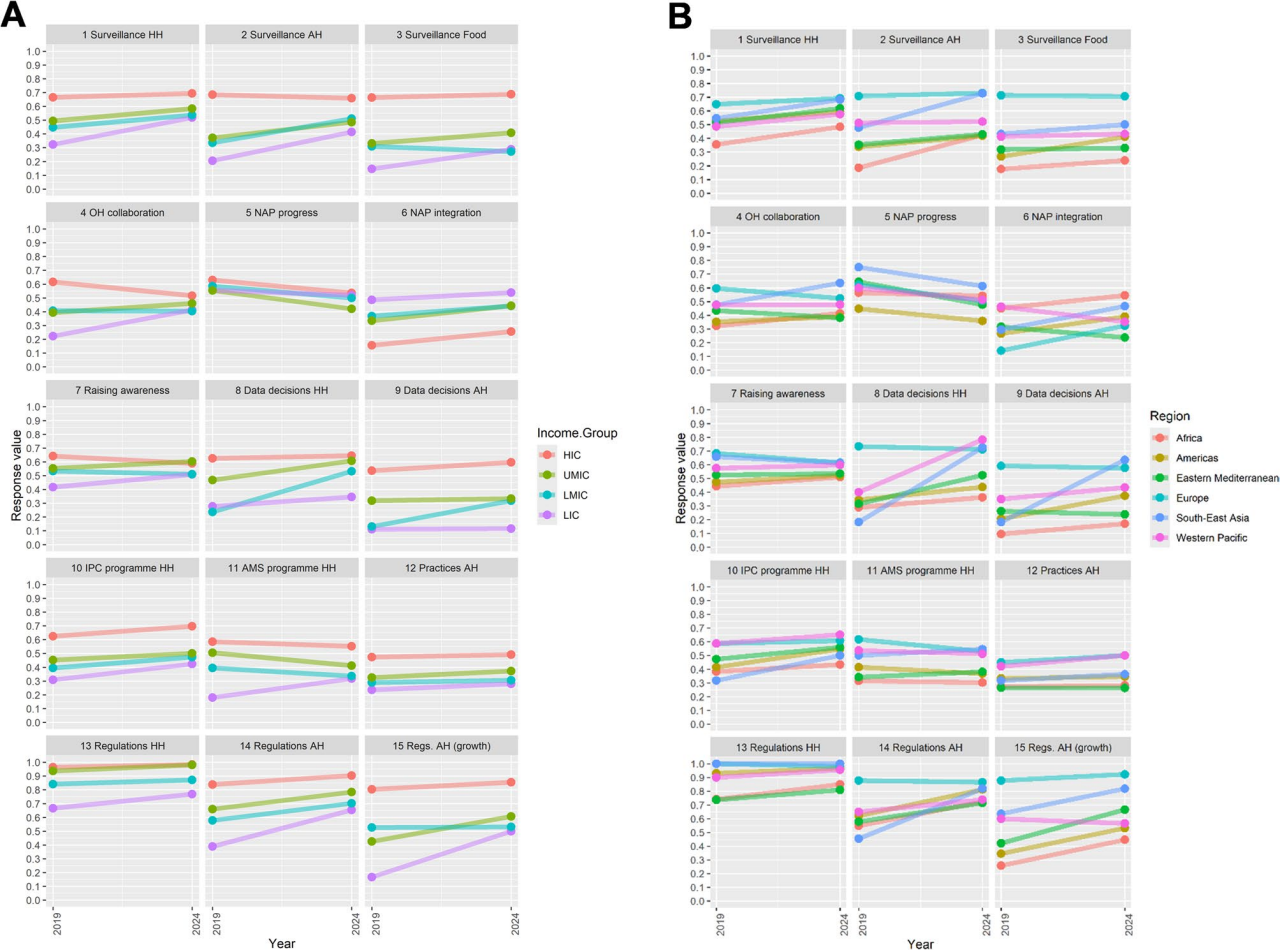
Slope graph showing country topic change over period, by **A**) income level (left) and **B**) region (right)

The slopegraphs in Fig. 4 below show differences in change over the period 2019–2024, categorising countries by income level and region (panel A at left and B at right, respectively). Contrasting line slopes for any topics suggest diverging trajectories for groups of countries.

Panel A on the left side of Fig. 4 shows countries grouped by income level. Slopes appear fairly consistent overall. On some topics, country group slopes suggest a consistent slight upward trend (NAP integration, IPC programme HH, Practices AH – topics 6, 10 and 12). NAP progress was the only topic showing a consistent downward trend (topic 5).

There is some apparent tendency, in other topics, towards lower-income countries closing the gap with high income countries (HIC). The spread of income level group response means is generally tighter in 2024 than in 2019. On some topics, all country responses improved but lower-income country responses improved faster (for example, Surveillance HH- topic 1 and Regulations AH- topic 14). On other topics, convergence also involved HIC responses declining slightly (Surveillance AH and Raising awareness – topics 2 and 7). There was a relatively sharp response decline for HIC on OH collaboration (topic 4).

One recurring pairwise contrast between groups is between low-income countries (LIC) and Lower-middle-income Countries (LMIC). On various topics (topics 3, 4, 7, 11, 14 and 15) , LIC mean responses improved at a faster rate than LMIC. On some others, notably topics 8 and 9, LMICs’ response improvement outstripped LICs’.

Panel B on the right side of Fig. 4, shows countries groups by WHO region. Slopes again appear fairly consistent between country groupings with some exceptions.

European countries’ responses were markedly better than other regions’ on various topics in 2019, including on Surveillance AH and Food (topics 2 and 3), Data decisions HH and AH (topics 8 and 9) and topics concerning AH regulatory frameworks (topics 14 and 15). By 2024, European countries’ responses were only exceptionally positive in Surveillance Food (topic 3).

South-East Asia exhibits particularly strong progress across the period on some topics, including Surveillance AH (topic 2), OH collaboration (topic 4), Data decisions HH and AH (topics 8 and 9) and Regulations AH (topic 14).

Western Pacific countries’ response trends also contrast with other regions on a couple of topics, positively in the case of Data decisions HH (topic 8) but negatively in the case of Regulations AH (growth promotion) (topic 15).

### Stage 2A: Change score differences between FF and other countries

Comparison of change scores between FF-supported countries and other countries suggested notable differences on some topics, with a possible causal relationship to programme interventions.

**Fig. 5:**
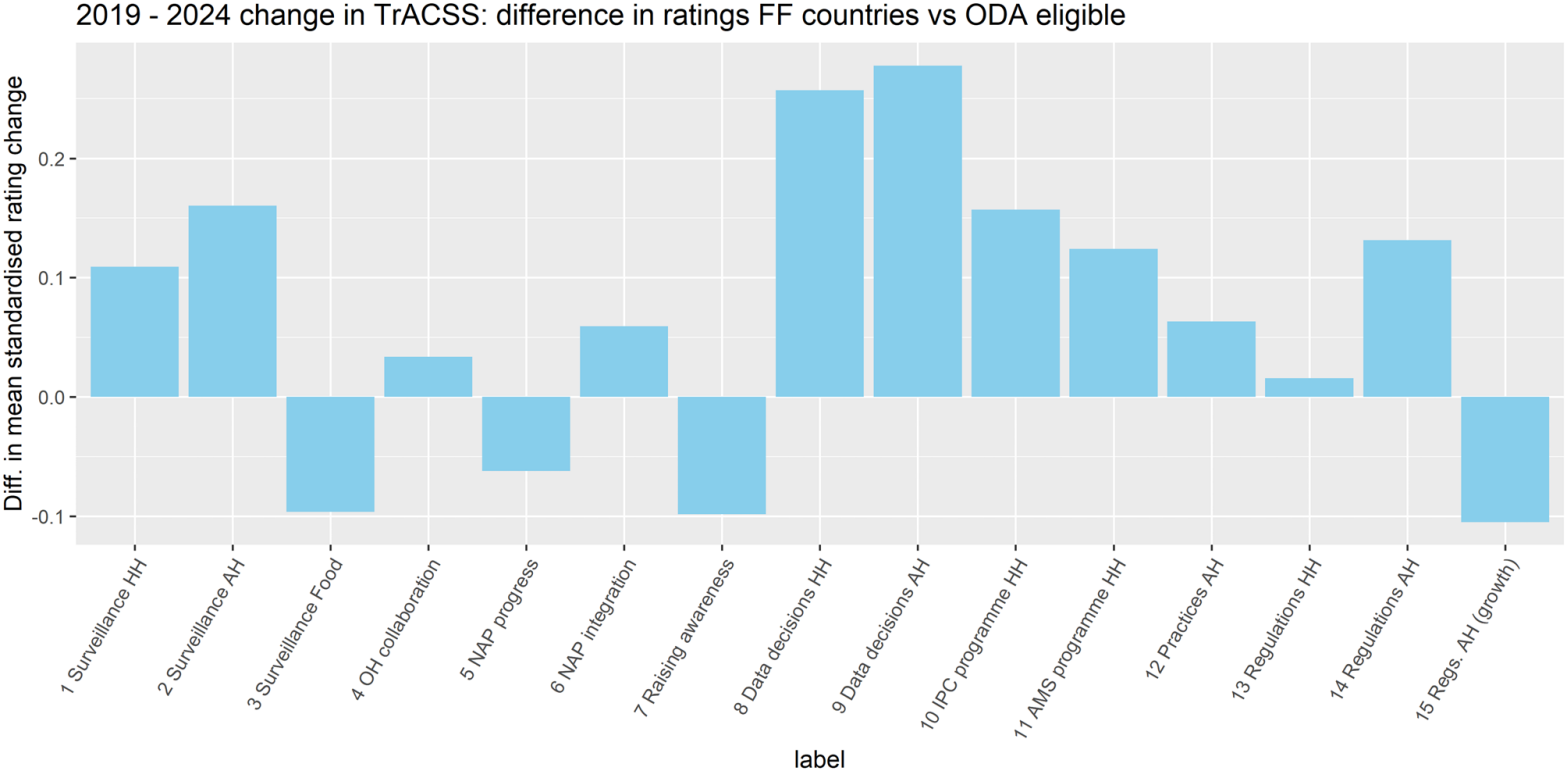
Change scores of FF-supported countries vs other ODA-eligible countries

Figure 5 below shows the difference between FF-supported countries and other ODA-eligible countries. In this comparison, ‘data decisions’ in the sense of review of AMR data when considering strategy appears to have progressed relatively strongly in FF countries across both HH and AH (topics 8 and 9). Relative progress is also apparent in:

- Surveillance in HH and AH (topics 1 and 2).
- IPC and AMS programmes in HH (topics 10 and 11).
- Regulations in AH (topic 14).

However, no distinct relative positive change, and in some instances negative differences, are apparent in other topics, notably:

- Surveillance in Food (topic 3).
- OH collaboration, NAP progress and NAP integration (topics 4-6).
- Raising awareness (topic 7).
- Regulations in HH (topic 13).
- Regulations in AH (for growth promotion) (topic 15).

It was not possible to calculate change scores for two FF-supported countries (Eswatini & Senegal), due to a lack of TrACSS response from these countries in 2019.

It proved difficult to obtain a reliable control group of countries not supported by FF for comparison purposes. FF-supported countries are mainly LMIC (18 out of 22), but with some LIC (3 – Malawi, Sierra Leone, Uganda) and one upper middle-income country (UMIC) (Indonesia). They are concentrated in the Africa and South-East Asia WHO Regions, but with some in Western Pacific (Lao People’s Democratic Republic, Papua New Guinea, Viet Nam) and one in Eastern Mediterranean (Pakistan). Table 3 shows score differences across all study topics for the main control groups considered, with considerable variation evident between change score differences depending on control group.

**Table 3:**
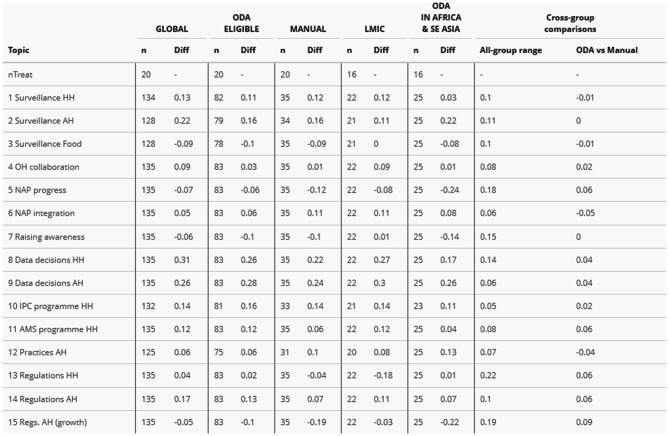
Differences in change scores between FF-supported countries and main control groups

The global comparison (FF-supported countries vs all others) was considered unsuitable due to the inclusion of HIC in the control group. As observed at Stage 1 of the analysis, HIC countries tended to exhibit different trajectories, with lower change scores tending to boost estimations of FF effect. On the other hand, focusing on smaller control groups (LMIC only, or ODA-eligible countries in Africa and South-East Asia only) carried the disadvantage of reducing the number of FF countries included to only 16 countries (excluding 4, or 20%), as well as reducing the size of the control group and therefore the power of comparison. The manual comparison group (see Fig. 1 in Methods) lacked rigorous definition, being more of a pragmatic, intuitive approach. However, change score differences with the manual control group were quite similar to those with ODA eligible countries. One advantage of ODA eligibility is that it is a relevant criterion for country selection, since FF is a development aid programme. ODA eligibility was settled on as a ‘least-worst’ option.

### Stage 2B: Significance testing association of score differences with FF intervention variables

Using statistical tests to consider the significance of score differences based on FF intervention variables indicated a strong relationship between FF interventions and improved change scores for three study topics. The results of the tests conducted are reported in Table 4 below. This indicates a statistically significant association between FF intervention variables and change scores at a 95% confidence level for:

- Surveillance in HH (topic 1).
- Data decisions in AH (topic 9).
- IPC programme in HH (topic 10).

Statistically significant association at a 90% confidence level is also apparent for:

- Surveillance in AH (topic 2).
- Data decisions in HH (topic 8).

**Table 4:**
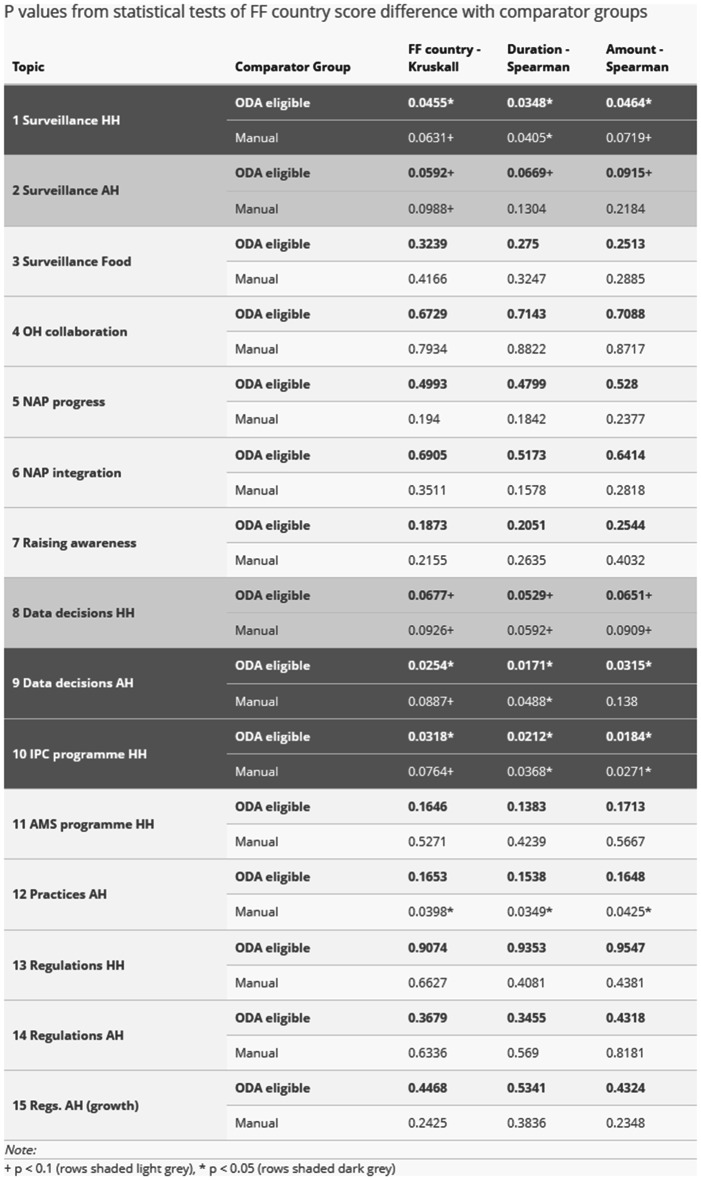
Statistical tests of differences in change scores

Duration of FF support appeared to be generally slightly more strongly associated with change score differences than other measures of FF support (binary country identifier or amount of FF funding). None of the topics on which there was a negative change score difference between FF-supported and other ODA-eligible countries exhibited a statistically significant association.

### Stage 2C: Regressions testing stage 2B results

Table 5 shows the outputs of regression models constructed for this stage of the analysis. Estimates of FF support variable effect on mean change scores were not large and the relationship was not shown to be statistically significant in any of the three topics considered.

**Table 5:**
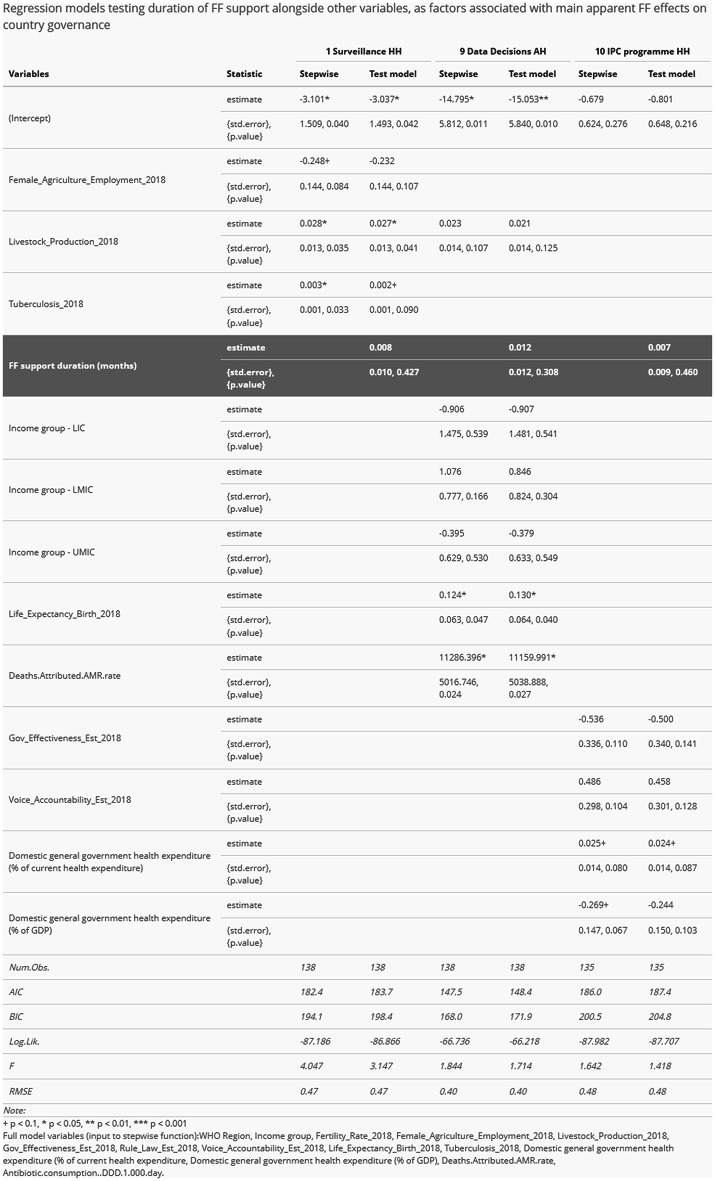
Regression modelling outputs

Final selection of variables for inclusion in original full models is as listed at the bottom of Table 5. Some variables were excluded on the grounds of multicollinearity, for example country governance indicators. Other variables were excluded on the grounds of no apparent correlation with dependent variables, for example GSEAR data on ‘political commitment’.

In the case of Surveillance in HH (topic 1), it proved difficult to model for country change scores using the available data. Stepwise modelling indicated that a livestock production index and estimates of tuberculosis incidence were the most influential factors in predicting relevant change scores over 2019–2024. High intercept estimate and p-values suggested poor model validity.

In the case of Data decisions in AH (topic 9), HIC status – as indicated by the intercept values – appeared to be the most influential (negative) factor. It was also notable that AMR mortality estimates appeared in stepwise model output.

IPC programme in HH (topic 10) was the only model output in which governance and health expenditure factors featured in stepwise model outputs. Expenditure appeared to be the most important category of factors in that case, with domestic as a proportion of overall spending apparently the single most influential factor.

## Discussion

### Objective 1: Assessing progress under the global action agenda on AMR

Our overall observation from stage 1A of limited positive global change is consistent with existing research in that it points to lack of general transformative change in AMR governance over recent years. Global reported progress was weak. Perhaps as to be expected, given the disruption caused by the COVID-19 pandemic during these years [39], it would be hard to claim that TrACSS reporting suggests a major governance breakthrough of any kind.

One issue from stage 1A is that reporting on NAP progress (topic 5) may indicate little gain because the response grades became more challenging for 2024 than they had been in 2019. NAP progress is an anchor topic for research on AMR governance, as noted in the Background. This finding suggests a need for future work making comparisons across TrACSS years to take special care over factor consistency.

Results from stage 1B offer more of a contrasting view with existing research. Positive global changes derived largely from the convergence of lower-income countries with HIC (Fig. 4A), and from other regions’ reporting with Europe’s (Fig. 4B). As noted in the Background, the prevailing view is that progress on AMR governance is proving especially difficult in lower-income countries, mainly because of resource constraints. The view from this study is more that progress has proved more difficult in HIC and in Europe, possibly because of higher 2019 baselines and less room for improvement. One opinion paper has advanced the view that ‘some progress is being made in implementing surveillance measures’ in low- and middle-income countries [40]. Our results enlarge on this suggestion.

Topic-wise, changes in TrACSS reporting over 2019 to 2024 indicates positive global progress across three topic pairs in particular:

- HH and AH surveillance systems themselves (topics 1 and 2). Setting aside wider questions about governance, for HH at least this would align with observations from global AMR surveillance such as country participation in the WHO Global Antimicrobial Resistance and Use Surveillance System (GLASS) reporting system [41]. The positive trend is harder to explain in AH. AH governance progress is normally seen to be harder given the larger role of the private sector in AH [12].
- HH and AH data decisions, responses to questions about the use of AMR surveillance data for decision-making and changes to strategy (topics 8 and 9). It is worth noting that these two topics have low factor consistency across 2019 and 2024, making it hard to judge the practical significance of apparent global absolute changes between the two years (see Table 1 and Technical Annex). However, advances in surveillance systems (topics 1 and 2 above) could help explain some positive changes in ratings for these topics, possibly mitigating lack of data as a basic constraint.
- AH regulatory frameworks (topics 14 and 15). Over this period, AH regulatory frameworks reportedly advanced to a level comparable with HH. Nearly 20% of countries also reported taking action to prohibit use of antibiotics for growth promotion (Fig. 3). Regulatory frameworks are an explicit focus of Quadripartite efforts under the World Organisation for Animal Health (WOAH) to promote standards at country level, including on growth promoters. They are also supported by the FAO AMRLex regulatory database, introduced in 2022 [42, 43]. Agricultural AMU was also a major focus of late 2024 UN meetings [44]. Our results might be understood as evidence confirming global progress from those efforts.

One of the most striking observations in our results from Stage 1B, about South-East Asia exhibiting particularly strong progress, holds true across each of these three topic pairs. This tends to bear out the detailed claims about proactive WHO regional office attention to the issue of AMR, including mentions of heightened perceptions of AMR risks in the region and firm political engagement by countries in regional cooperation around AMR [45]. An interesting ‘One Health-based system-wide evaluation tool on global AMR’ suggested Malaysia and Thailand as early-mover countries on AMR governance [46]. However, explanations of why and how South-East Asia might have become a strong example in terms of country governance of AMR surveillance are not clear in the wider literature.

There are some interesting distinctions from within the overall observation on One Health collaboration (topic 4). Variations by income level and region are apparent including: declining responses for HIC (Fig. 4A); and contrasting trajectories of decline in Europe and positive change in South-East Asia (Fig. 4B). Possibly this could support the idea that OH has been implemented differentially outside HIC, in more of a bottom-up way and thus less vulnerable to bureaucratic distractions [47].

NAP integration (topic 6) is the only topic on which there is an inverse relationship apparent between response and income level. In other words, NAPs are reportedly better-integrated in poorer than in richer countries (Fig. 4A). These findings, including regional contrasts, may reflect assumptions in the TrACSS questions about the formal appearance of integration. Any interpretations of results on this topic should consider the detail of issues with low factor consistency on this topic (see Technical Annex).

Our global results on raising awareness (topic 7) suggest that national awareness campaigns have not tended to progress much globally, with reported gains in some countries accompanied by decreases in reported activity levels in others (Fig. 3). Including an indication of possible activity decreases is an interesting reflection on studies of awareness campaigns, notably a survey of relevant research over 2010–2022 which suggested greater activity in HIC [48].

Another topic on which little global progress appears in our results is AMS programmes in HH (topic 11). It is difficult to sense-check this result against existing evidence because of a lack of research on national AMS programmes. AMS is usually addressed at healthcare facility level [49, 50]. Although WHO has issued relevant guidance, the limited studies addressing AMS at the country level would be consistent with an estimate of limited progress [51–53]. WHO Access, Watch, Reserve (AWaRe) guidance is normally understood to have been the main mechanism for global agenda influence on country AMS [54]. AWaRe adoption has been addressed by a separate TrACSS question since 2020 [24], so is not included in this study.

Overall, the results of this study suggest that the influence of the global action agenda on country governance may be under-estimated in current literature. While it is reasonable to conclude that progress in terms of NAP implementation has been slow [9, 13], there are other aspects which appear to have met with more success. Looking at country governance of AMR surveillance, the global agenda appears to involve lower-income countries in regions outside Europe reporting increasingly positive developments. AH regulation stands out as one topic on which global standards are exerting influence at country level.

From this perspective, criticisms of NAPs’ lack of substance [16] may tend towards over-expectation about how global governance mechanisms influence action. UN systems engage in policy influence activities variously across its component organisations, but almost always in a supportive, technical role confined to formulation of normative standards [55] rather than in terms of instrumental substance under a distinct set of plans.

Considering the potential implications of global agenda progress for country-level AMR surveillance efforts, relatively strong agenda positioning in the regulatory domain may be one useful point for deliberation. This suggests that AMR action agenda efforts might usefully focus on comparisons with agendas placing relatively strong emphasis on regulatory modes (such as tobacco control) [56]) and to research strands addressing global efforts to situate AMR in international law [57, 58].

### Objective 2: Assessing FF effectiveness as a major development aid initiative on AMR surveillance

The results of this study add to existing evidence of FF effectiveness in terms of country governance of AMR surveillance. Above all, from stage 2B of our analysis there is a strong association apparent in ODA-eligible countries between FF intervention and higher 2019–2024 change score differences on:

- Topic 1, Surveillance HH (at 95% confidence level).
- Topic 2, Surveillance AH (at 90% confidence level).

These findings are consistent with existing evidence from FF programme evaluation [19]. Surveillance system strengthening is a core aim in the FF Theory of Change (see Technical Annex) and our results in this regard find ready explanation in FF activity reporting.

Our analysis stage 2B results also show a similarly strong association on:

- Topic 8, Data decisions HH (at 90% confidence level).
- Topic 9, Data decisions AH (at 95% confidence level).

In this case, our findings are again qualified by the observation that topics 8 and 9 suffer from relatively weak factor consistency between 2019 and 2024. This issue is arguably less serious for cross-group than absolute estimates. However, the findings are also inconsistent with existing evidence. Relevant TrACSS questions cover two aspects (see Technical Annex): processing of relevant information (‘reviewing’ or generally ‘using’ data); and then taking relevant action (‘amending’ strategy or policy, or simply ‘informing’ operational decisions). Independent evaluation in 2023 tended to confirm that processing is taking place in FF countries, but also reported difficulties in identifying actions that have been taken as a result [19]. Ideally, our observation from TrACSS on these topics should be linked to concrete examples of action.

Across both surveillance system and decision-making topics, FF effectiveness is indicated in AH as well as HH. As mentioned above (Discussion – Objective 1), AMR surveillance in AH is normally regarded as more challenging than in HH. Broadly speaking, evidence of effectiveness across AH as well as HH is reflective of FF’s stated ambition to operate as a One Health programme.

A final positive finding from stage 2B of the analysis is another strong association of this type, at 95% confidence level, on Topic 10: IPC programme HH. This result is novel and defies ready explanation beyond FF targeting of IPC outcomes at impact level in the Theory of Change. To the extent that the finding indicates real underlying change in country systems, this would represent a major programme achievement. Further investigation is required for clarification of possible causal links.

The practical significance of each of these estimated FF effects across countries were substantial despite potentially not appearing transformative. On data decisions, FF-supported countries were approximately a quarter (25%) more likely to respond ‘yes’ than would be expected otherwise. On the other topics, including HH and AH surveillance systems strengthening, about half (50%) of FF-supported countries were one grade higher on the relevant TrACSS response scale than would be expected otherwise.

There are four topics on which the results of this study indicate that FF may be obtaining negative effects. Although none were found to be statistically significant, it is at least notable that our results do not support the suggestion of FF achieving any positive effects on any of these areas. The relevant topics are:

- Surveillance Food (topic 3).
- NAP progress (topic 5).
- Raising awareness (topic 7).
- AH regulations for growth promotion (topic 15).

None of these topics were included in the explicit objectives for FF interventions at country level, so it is important to observe that lack of evidence for FF effectiveness in these areas should not be mistaken as evidence for ineffectiveness. Some of these topics may be of interest to AMR-related programmes at the strategic level, however. Given the discontinuation of FF in 2026, it is too late for the programme to take our results into account. But our results might suggest value to other programmes of finding better connections between global investments and dynamics, on the one hand, and country-level interventions, on the other.

Our results from Stage 2 analysis are best understood as a partial estimate of FF results overall. The overall approach is justified because governance interventions at country level through partnership with lower- and middle- income countries remained FF’s core activity and therefore a crucial dimension of questions about programme effectiveness. Building on investments in microbiology laboratory capacity for these purposes, FF set out to support countries in implementing the surveillance component of their NAPs including through initiatives designed to overcome governance obstacles [59, 60]. However, FF funded global as well as regional initiatives, including direct grants to Quadripartite organisations for AMR-related activities. Global or other results from these activities might tend to create a general difference effect at country level, leading to under-estimation from our chosen methods for Stage 2 of the analysis (less apparent difference in differences).

Our results from Stage 2 analysis are also only approximate estimates because of difficulties splitting out FF from other interventions. Some instances of FF global initiatives, notably the Multi Partner Trust Fund (MPTF), involved co-funding AMR aid activities with other actors [61]. Although FF stood out as the leading global programme focused on AMR surveillance systems development, a wide range of other relevant development aid initiatives were also relevant, for example the Global Antimicrobial Resistance Partnership (GARP) and the Medicines, Technologies, and Pharmaceutical Services (MTaPS) Program [62, 63].

Null results from stage 2C of the analysis do little to undermine the value of results from stage 2B, although they certainly do not strengthen our findings. This aspect of our study serves as a useful reminder that the factors under consideration remain imprecise and difficult to explain. Looking to the results in Table 5, sense-making is difficult beyond possibly the apparent importance of health system expenditure for the stepwise model output for topic 10 (IPC programme HH). It is reasonable to expect, even qualitatively on the grounds of wider AMR-related development aid programming, that the significance of FF results may recede when considered relative to other factors.

### Reflections on the weaknesses and strengths of TrACSS data

The TrACSS system is itself an integral part of the governance systems being studied: TrACSS responses are themselves expressions of country AMR governance; the survey is administered by the WHO under the GAP; and according to FF’s website the effort is funded by the UK through FF.

The main weakness of TrACSS data is reliability. Although the data has been frequently used for AMR governance and policy research, it is usually accompanied by notes on limitations on its nature as unverified self-assessment data with clear risks of unreliability due to social desirability bias or mis-estimation [13, 38].

TrACSS could ideally be triangulated with expert assessments, possibly following the model established for the States Parties Self-Assessment Annual Reporting (SPAR) tool for reporting under the International Health Regulations (IHR). In that case, concerns about reliability led to the creation of the Joint External Evaluation (JEE) exercise [64]. However, SPAR continues as a best-practice source for IHR assessments in the interim between less-frequent JEE exercises [65]. There may also be opportunities for TrACSS triangulation with information from GLASS systems.

Discussion above, especially on NAP progress (topic 5), suggests that lack of factor consistency over time is another major weakness in TrACSS data, undermining utility for longitudinal analysis purposes and potentially underestimated in research to date relative to reliability issues.

TrACSS also conditions nation states as the unit of analysis. National governments play a crucial role in AMR governance, and TrACSS questions focused on the country level often speak to other system levels (especially local elements as components of health systems – see Technical Annex). Still, this remains a significant limitation of TrACSS data.

On the other hand, TrACSS has some strengths which are not well-rehearsed in current research. Data becomes available relatively quickly which enables production of more current assessments than are possible using other sources. The system now has high country participation rates (186 for 2024) which enables comprehensive global assessments and extensive cross-country comparison. With the passage of years, the data also gains value in terms of longitudinal persistence. TrACSS data originates from national governments and is therefore in some sense ‘owned’ by countries. The data is also publicly available which makes it suitable for open research. And it can be reasonably assumed to be low-cost to produce, at least relative to the expert appraisal mechanisms currently considered as the main alternative.

Referring to broader experience from research on social indicators [66], the value of TrACSS data ultimately depends on the paradigm being applied. From a positivist perspective, reliability concerns are potentially decisive. Interpretivist and constructionist paradigms are better equipped to account for indicators as a ‘technology of global governance’, with interesting but under-considered social dynamics around compilation, promulgation and influence [67].

For current purposes, applying a pragmatic approach, validity hinges on intended uses. Firstly, the fact remains that the TrACSS system was set up to monitor and encourage relevant country governance. Secondly, using global indicators like TrACSS to monitor global progress is less vulnerable to (and less likely to provoke) social desirability bias than other applications like performance appraisal or ranking [68] or decision-making about development aid priorities or targets [69]. Finally, TrACSS is currently the best-available resource to address a lack of evidence on FF programme effects at higher outcome level. There is a general concern for evaluation of policy-related approaches that evidence on *impacts*^4^ tends to be overlooked.

### Study limitations

No effort was made to triangulate or otherwise validate TrACSS data as part of the analysis for this study. Data reliability issues may partially explain observed results, for example countries benefiting from aid support (such as FF) may have incentives to report improvements aligned with expectations. This is a considerable limitation given understandings of the unreliability of this data source, but justified in the context of the pragmatic approach. Future initiatives could helpfully focus on this issue, for example drawing on JEE data for research as noted above or on data from the FF programme itself for evaluation.

Compromises were made in some instances about the level of factor consistency required between 2019 and 2024 questionnaire responses to represent change over time on some topics. These issues were flagged using factor consistency grades in Table 1 and full details in the Technical Annex.

Representation of TrACSS responses over time as difference between two discrete survey rounds after a 5-year interval are misleading to the extent that they suggest relevant governance topics in terms of simple linear change. Changes in responses would ideally be captured over all relevant years, providing more insight into the way changes have been reported year by year.

Issues of effectiveness delay – time taken for true effects to materialise – are also relevant here (for example, relative HH AMR surveillance reporting levels may continue to strengthen over coming years, or apparent relative gains may recede if there are issues sustaining relevant change).

The intended elaboration of difference-in-differences methodology was disrupted by difficulties in establishing a clear control group, as discussed in the Results for stage 2A. Any future evaluation efforts working along these lines could helpfully anticipate and mitigate these issues more effectively.

Regression modelling for stage 2C of the analysis was very limited in scope. There is potential, subject to further preparations, for further regression modelling using study data to pursue other objectives, including explanations for the state of TrACSS responses (instead of change over time) in terms of wider country indicators.

## Conclusions

This study addresses country governance of AMR surveillance as a focus in global governance and development aid discussions, drawing on a specific dataset. It should not be taken in isolation as a reliable comprehensive assessment of relevant social dynamics. Ultimately, study results are best understood as a research contribution to ongoing policy conversations about progress through the global action agenda on AMR and strategic orientation of a major AMR surveillance development programme as part of that agenda. Our study adds to current literature by making pragmatic empirical observations about country governance systems as they relate to AMR surveillance efforts; as AMR surveillance efforts mature, gaps have emerged in research about the governance systems involved.

In terms of the global action agenda, this study challenges current narratives about AMR governance progress being especially difficult and slow in lower-income countries. Reporting on surveillance-related topics confirms that governance remains less developed in lower-income countries than in higher-income ones. But considering change over time from 2019 to 2024, it is mostly lower-income countries that have been making progress on AMR country governance of AMR surveillance. There is a sense of progressive momentum, above all in South-East Asia, disrupting established perceptions that European countries lead the world in AMR surveillance.

In terms of development aid effectiveness, this study provides concrete, quantitative comparative evidence of FF impacts in terms of wider country systems. Using TrACSS data enables quantitative estimates of programme results based on comparisons with other countries. Country reporting through TrACSS in 2019 and 2024 indicates that the UK Fleming Fund has not only strengthened HH and AH surveillance systems in partner countries but has likely also achieved concrete institutional changes around the use of relevant data in administrative decision-making processes. The data also shows indications of emergent positive change on other Fleming Fund objectives, including possible translation of surveillance system gains and other interventions into effects in terms of policy, programmes and regulation.

While these are useful results against each of the study’s objectives, this paper also suggests grounds for critical reflection and a need for further work on the country governance of AMR surveillance. Contrasting views from different research perspectives about the exact scope of surveillance efforts and their relationship to country governance indicates a need for more work to establish common ground. Observations about apparent change over 2019–2024 help emphasise shared experience and inform planning for future efforts.

## Electronic supplementary material

Below is the link to the electronic supplementary material.

Supplementary Material 1

## Data Availability

Data and software used for this study have been made available through an Open Science Framework repository: https://osf.io/yp79a/?view_only=78066d971314454793219e983693efe8.

## Abbreviations

AH: Animal Health
AMR: Antimicrobial Resistance
AMS: Antimicrobial Stewardship
AMU: Antimicrobial Use
AMU/C: Antimicrobial Use and Consumption
AWaRe: Access, Watch, Reserve: WHO guidance on categories for AMU/C
CG: Country Grant
DHSC: Department of Health and Social Care (UK)
EH: Environmental Health
FAO: Food and Agriculture Organization of the United Nations
FF: Fleming Fund
GAP: Global Action Plan
GARP: Global Antibiotic Resistance Partnership
GLASS: Global Antimicrobial Resistance and Use Surveillance System
GRAM: Global Research on Antimicrobial Resistance
GSEAR: Global Survey of Experts on Antimicrobial Resistance
HH: Human Health
HIC: High-income countries
IHME: Institute for Health Metrics and Evaluation
IHR: International Health Regulations
IPC: Infection Prevention and Control
JEE: Joint External Evaluation
LIC: Low-income countries
LMIC: Lower-middle income countries (wider sense of all low- and middle-income countries is spelled out)
MPTF: Multi Partner Trust Fund
MTaPS: Medicines, Technologies, and Pharmaceutical Services
NAPs: National Action Plans (on AMR)
ODA: Official Development Assistance
OECD: Organisation for Economic Co-operation and Development
SPAR: States Parties Self-Assessment Annual Reporting
TrACSS: Tracking AMR Country Self-Assessment Survey
UK: United Kingdom
UMIC: Upper-middle income countries
UN: United Nations
UNEG: United Nations Evaluation Group
UNEP: United Nations Environment Programme
WAAW: World AMR Awareness Week
WHO: World Health Organization
WOAH: World Organisation for Animal Health (formerly OIE)

## Footnotes

1 Recognising the necessity of a ‘One Health’ approach to cross-sectoral collaboration on AMR, amongst other global health challenges, the global action agenda is currently being pursued by the ‘Quadripartite’ of global governance organisations: the Food and Agriculture Organization of the United Nations (FAO), United Nations Environment Programme (UNEP), World Health Organization (WHO) and World Organisation for Animal Health (WOAH).

2 The other GAP objectives are: communication, education and training; development of the economic case for sustainable investment; antimicrobial stewardship (AMS); and infection prevention and control (IPC).

3 Relevant countries (WHO regions, WHO income classifications): Bangladesh (SEARO, LMIC), Bhutan (SEARO, LMIC), Eswatini (AFRO, LMIC), Ghana (AFRO, LMIC), Indonesia (SEARO, UMIC), Kenya (AFRO, LMIC), Lao People’s Democratic Republic (WPRO, LMIC), Malawi (AFRO, LIC), Myanmar (SEARO, LMIC), Nepal (SEARO, LMIC), Nigeria (AFRO, LMIC), Pakistan (EMRO, LMIC), Papua New Guinea (WPRO, LMIC), Senegal (AFRO, LMIC), Sierra Leone (AFRO, LIC), Sri Lanka (SEARO, LMIC), Timor-Leste (SEARO, LMIC), Uganda (AFRO, LIC), United Republic of Tanzania (AFRO, LMIC), Viet Nam (WPRO, LMIC), Zambia (AFRO, LMIC), Zimbabwe (AFRO, LMIC).

4 An ‘intervention’s ultimate significance and potentially transformative effects – holistic and enduring changes in systems or norms’ [70].

## Glossary

**Surveillance**: Public health surveillance aims to provide information to support timely, actionable information to those responsible for relevant actions [71].
**Governance**: Governance is the complex, multidimensional set of processes through which responsibility is distributed amongst health system actors [72]. It is a concept often elaborated in global health frameworks establishing expectations for country system improvements, as in the WHO *Building Blocks* for example [73].
