# Supplementary material for: Country governance of antimicrobial resistance (AMR) surveillance: observations on global progress and aid programme effectiveness using data from the Tracking AMR Country Self-Assessment Survey (TrACSS)
Source: Global Health. 2026 Jan 7;22:8. doi: 10.1186/s12992-025-01179-4 (PMC12822036; doi:10.1186/s12992-025-01179-4)
Supplement: Supplementary file 1 — Supplementary Material 1 [file 12992_2025_1179_MOESM1_ESM.docx]

**TECHNICAL ANNEX**

# FF Theory of Change

As explained in the manuscript, topic selection was undertaken based on relevance to the FF Theory of Change (see below excerpt from the FF 2021 Annual Review as referenced in the text):


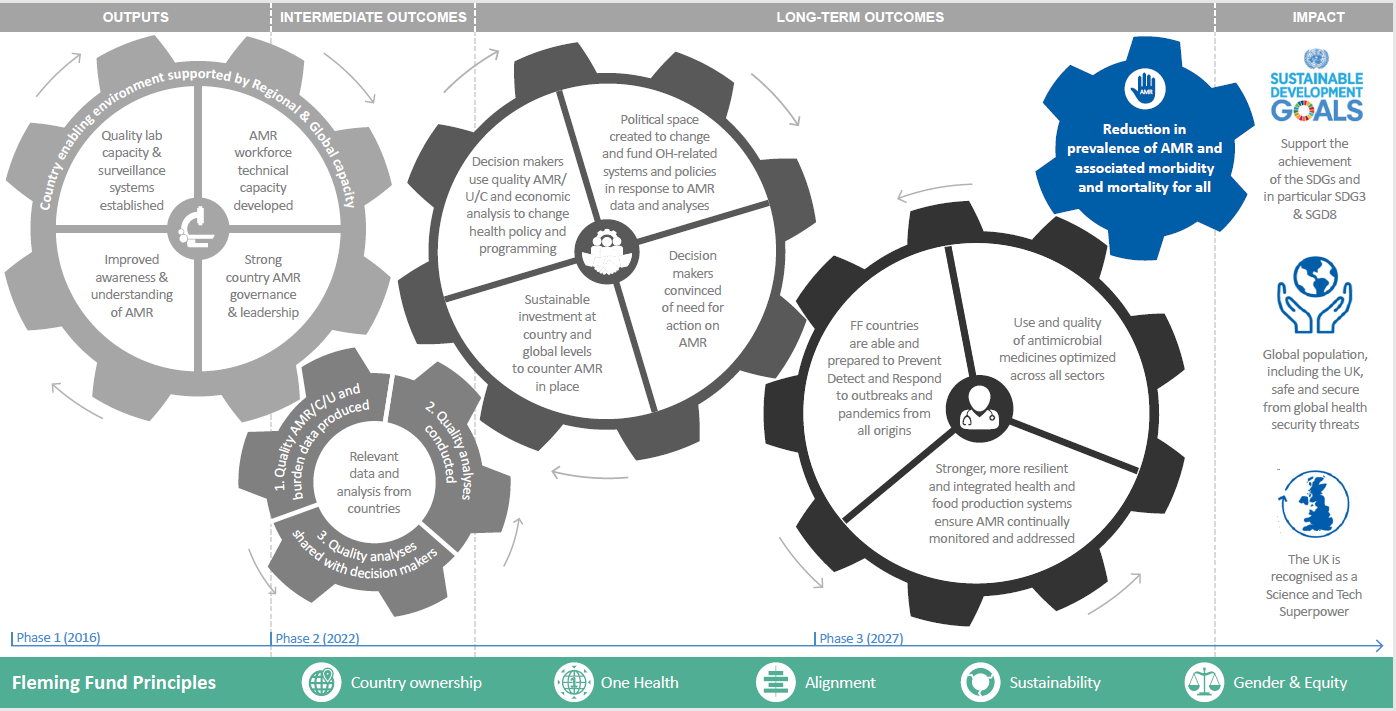


Broadly speaking, the Theory of Change describes a causal process over time (left to right) in which outputs from surveillance systems themselves are expected to encourage analytical and administrative processes, strengthening a country’s resolve to act in various ways.

# Topic groups

Study topics were arranged into groups (as shown in Table 1):

- ‘Monitoring’ topics addressed development of underlying national lab-based AMR surveillance systems (including intended results for FF at output and intermediate levels).
- ‘Organising’ topics addressed key concerns in the AMR governance literature around OH collaboration and NAP development (not explicitly linking to the FF Theory of Change but reflecting assumptions output connections to outcomes).
- ‘Understanding’ topics reflected two rival approaches to outcome-level results generation, one targeted by FF (administrative decision-making) and the other not directly prioritised (communications campaigns for public awareness).
- ‘Mobilising’ topics included key domains for intended policy action to counter AMR: antimicrobial stewardship (AMS), infection prevention and control (IPC) and AH practices. Although relevant to various aspects of the FF Theory of Change, these topics relate most closely to higher outcome results levels.
- ‘Regulating’ topics included key domains for intended regulatory framework elaboration in country action against AMR. Again, these relate most closely to higher outcome results levels in the FF Theory of Change.

# Response scales, numeric values, scoring & standardisation

This section outlines the methodology used to process and standardize responses from the 2019 and 2024 TrACSS survey data. It details the treatment of varying question formats, and the scaling techniques applied to enable comparison across years and groups.

The surveys included questions with different response scales, primarily a 5-point scale (presented as an A-E format) and a Yes/No format. A summary of the response scales and conversions used across the 15 study topics is presented below.

| **Number** | **Study topic** | **Response scale** | **Numeric conversion** | **Standardisation to 0-1 scale** |
| --- | --- | --- | --- | --- |
| 1 | Surveillance HH | A-E | 0-4 | ÷4 |
| 2 | Surveillance AH |  |  |  |
| 3 | Surveillance Food |  |  |  |
| 4 | OH collaboration |  |  |  |
| 5 | NAP progress |  |  |  |
| 6 | NAP integration | 4 non-exclusive options (2019)  8 non-exclusive options (2024) | +0.25 per option (2019)  +0.125 per option (2024) | NA |
| 7 | Raising awareness | A-E | 0-4 | ÷4 |
| 8 | Data decisions HH | No/Yes | 0/1 | NA |
| 9 | Data decisions AH |  |  |  |
| 10 | IPC programme HH | A-E | 0-4 | ÷4 |
| 11 | AMS programme HH |  |  |  |
| 12 | Practices AH |  |  |  |
| 13 | Regulations HH | No/Yes | 0/1 | NA |
| 14 | Regulations AH |  |  |  |
| 15 | Regulations AH (growth) |  |  |  |

## 5-level scale responses

9 of the 15 study topics involved TrACSS questions with 5 levels of response options, marked with letters from A (indicating the lowest or null option) to E (indicating the most advanced option).

Responses captured on the 5-point scale were converted into numeric values, assigning integers from 0 to 4 (A = 0, B = 1, C = 2, D = 3, E = 4). Change scores were computed as the 2024 numeric score minus the 2019 numeric score.

Values for these topics were standardized by dividing the difference by 4 to obtain a range of 0-1 for responses and ±1 for change scores.

The following diagram provides a visual summary of this approach.

## Yes/No scale responses

5 of the 15 study topics involved TrACSS questions effectively requesting a Yes or No answer, although 3 of these were special cases requiring more detailed explanation (see below).

Responses to these questions were converted to numeric values, with "Yes" coded as 1 and "No" as 0. Change scores were computed as the 2024 numeric score minus the 2019 numeric score.

No standardisation was required for these topics since the range for responses was already 0-1 and for change scores ±1.

This approach is visualized in the diagram below.

## Special case: topic 6 (NAP integration)

Topic 6 (NAP integration) is a special case because relevant TrACSS questions suggested non-exclusive options for selection by respondents. In 2019, there were 4 possible options of subject areas that respondents could select as being linked to the NAP. In 2024, there were 8 specified plan types with which national planning on AMR could be integrated. See full details for topic 6 in the underlying responses section below.

The approach taken to numeric conversion of responses in this case was to add a certain value increment to a base value of 0 for each option selected: in 2019, 0.25 was added to the value for each option selected; in 2024, 0.125 was added to the value for each option selected.

No standardisation was required for these topics since the range for responses was already 0-1 and for change scores ±1.

This approach is visualized in the diagram below.

## Special case: topics 8 & 9 (data decisions – HH & AH)

Topics 8 (data decisions HH) and 9 (data decisions AH) were special cases of the Yes/No response approach explained above. This is because the 2024 questionnaire used two Yes/No questions to address a topic that was addressed by a single Yes/No question in 2019. See full details for topics 8 & 9 in the underlying responses section below.

The additional process followed for each of these topics was to use an ‘AND’ condition when assigning any positive numeric score for 2024 responses. That is to say, respondents needed to answer ‘Yes’ to both relevant questions in 2024 for the response to be assigned a value of ‘1’. Otherwise the numeric value of the relevant response remained ‘0’.

This approach was justified because it offered the strictest possible interpretation of the 2019 question, minimising the potential for 2024 scores to change over intervening time not resulting from some underlying change in behaviour. The approach is visualized in the diagram below.

## Special case: topic 14 (regulations AH)

Topic 14 (regulations AH) was another special case of the Yes/No response approach explained above. This is again because the 2024 questionnaire used two Yes/No questions to address a topic that was addressed by a single Yes/No question in 2019. See full details for topic 14 in the underlying responses section below.

The additional process followed for this topic was to use an ‘OR’ condition when assigning any positive numeric score for 2024 responses. That is to say, respondents only needed to answer ‘Yes’ one of the two relevant questions in 2024 for the response to be assigned a value of ‘1’. Otherwise the numeric value of the relevant response remained ‘0’.

This approach was justified on the basis of the wording of the 2019 question, which asked about ‘animals’ generally. Any relevant animal health regulation could have justified a ‘Yes’ answer in 2019. The approach is visualized in the diagram below.

## Interpretation

Observations about the strengths and weaknesses of the TrACSS data source are included in the body of the paper. However, it is worth bearing in mind that each round of data represents self-assessment data submitted by WHO member state governments and not subjected to any further validation process. Any unreliability in data for a year (2019 or 2024) is then potentially compounded when calculating change scores between years (2024 – 2019).

Standardisation of numeric value ranges, while it enables easier comparison of apparent response changes from 2019 to 2024, also masks persistent differences between study topics in terms of what different underlying response scales indicate for practical purposes. The following table is designed to aid interpretation of practical significance when considering change scores across groups of countries.

| **Topics** | **Scale** | **Interpretation of practical significance of change scores** |
| --- | --- | --- |
| 1-5, 7, 10-12 | A-E (0-4) | Change of 1 (or 0.25 when standardised) indicates one-grade shift on relevant TrACSS scale on average. |
| 8-9, 13-15 | No/Yes (0/1) | Change of 0.5 indicates average of one-half of countries changing their response. |
| 6 | Difference in proportion | General indication of change in degree to which country AMR plans associated with other plans considered by WHO to be relevant in given year. |

# Study topics: underlying responses and factor consistency

As noted elsewhere, TrACSS questions used to assess study topics did not stay consistent over time in all cases. Changes in scores may partially reflect differences in respondent understanding or interpretation of relevant questions.

Each study topic is presented in turn, setting out:

- A side-by-side comparison of questions and responses for 2019 and 2024 along with a Sankey diagram illustrating the flow of all responses across the two years
- An assessment of consistency in question framing and response options across the two years.

Factor consistency assessment considered changes in questions and response levels, ultimately combining these two considerations to estimate how likely it is that apparent response differences are caused by factor changes over time. Factor consistency levels were graded as follows:

| *Factor consistency level (see Table 1)* | *Likelihood that response differences caused by factor changes* | *Question changes* | *Response level changes* |
| --- | --- | --- | --- |
| **Full** | None | Identical | Identical |
| **High** | Some possibility  (but unlikely to be major influence) | Minor changes at most | Minor changes at most |
| **Moderate** | Plausible (probably playing a non-trivial role) | Significant changes but broad consistency in meaning | Significant changes including some effective re-categorisation |
| **Low** | Likely a major influence (or only general indication of change over time) | Major changes | Structural change to way questions addressed |

## 1 Surveillance HH

| **2019 QUESTION** | **SANKEY – ALL COUNTRIES 2019 - 2024** | **2024 QUESTION (tracked changes)** |
| --- | --- | --- |
| \| ***Question*** \| ***Response options*** \| \| --- \| --- \| \| 7.4 National surveillance system for antimicrobial resistance (AMR) in humans \| A- No capacity for generating data (antibiotic susceptibility testing and accompanying clinical and epidemiological data) and reporting on antibiotic resistance. \| \| B- AMR data is collated locally for common bacteria, but data collection may not use a standardized approach and lacks national coordination and/or quality management. \| \| C- National AMR surveillance activities for common bacterial infections follow national standards, and a national reference laboratory that participates in external quality assurance. \| \| D- There is a functioning national AMR surveillance system covering common bacterial infections in hospitalized and community patients, with external quality assurance, and a national coordinating centre producing reports on AMR. \| \| E- The national AMR surveillance system integrates surveillance of AMR across sectors, and generates regularly reports covering at least one common indicator. \| | 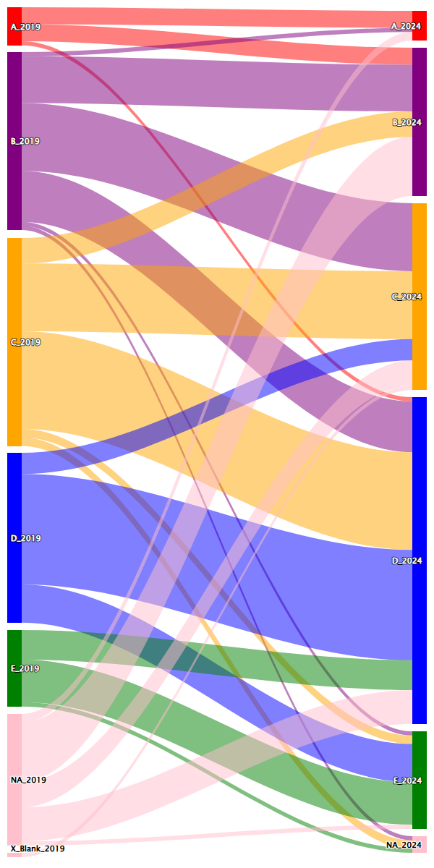 | \| ***Response options*** \| ***Question*** \| \| --- \| --- \| \| A- No capacity for generating data (antibiotic susceptibility testing and accompanying clinical and epidemiological data) and reporting on antibiotic resistance. \| 3.3 National surveillance system for antimicrobial resistance (AMR) in humans \| \| B- AMR data is collated locally for common bacterial infections in hospitalized and community patients, but data collection may not use a standardized approach and lacks national coordination and/or quality management. \| \| C- AMR data are collated nationally for common bacterial infections in hospitalized and community patients, but national coordination and standardization are lacking. \| \| D- There is a standardized national AMR surveillance system collecting data on common bacterial infections in hospitalized and community patients, with established network of surveillance sites, designated national reference laboratory for AMR, and a national coordinating centre producing reports on AMR. \| \| E- The national AMR surveillance system links AMR surveillance with antimicrobial consumption and/or use data for human health. \| |

| **FACTOR CONSISTENCY ASSESSMENT** |
| --- |
| **HIGH**  Identical question.  Minor changes to response levels.  Some possibility that response level changes may be causing apparent response differences (mention of national reference laboratory moved from C to D). |

## 2 Surveillance AH

| **2019 QUESTION** | **SANKEY – ALL COUNTRIES 2019 - 2024** | **2024 QUESTION (tracked changes)** |
| --- | --- | --- |
| \| ***Question*** \| ***Response options*** \| \| --- \| --- \| \| 7.5 (a) National surveillance system for antimicrobial resistance (AMR) in animals (terrestrial and aquatic) \| A - No national plan for a system of surveillance of AMR. \| \| B - National plan for surveillance of AMR but capacity (including laboratory and for reporting data on AMR) is lacking. \| \| C - Some AMR data is collected locally but may not use a standardised approach and lacks national coordination and/or quality management. \| \| D - Priority pathogenic/ commensal bacterial species have been identified for surveillance. Data systematically collected and reported on levels of resistance in at least 1 of those bacterial species, involving a laboratory that follows quality management processes, e.g. proficiency testing. \| \| E - National system of surveillance of AMR established for priority animal pathogens, zoonotic and commensal bacterial isolates which follows quality assurance processes in line with intergovernmental standards. Laboratories that report for AMR surveillance follow quality assurance processes. \| | 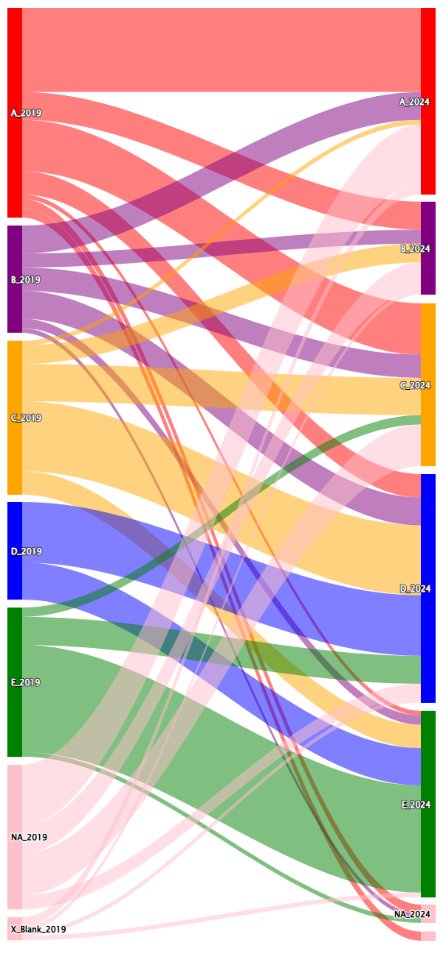 | \| ***Response options*** \| ***Question*** \| \| --- \| --- \| \| A - There are no local or national strategies/plans for generating AMR surveillance data from animals for an AMR surveillance system. \| 4.7 National surveillance system for antimicrobial resistance (AMR) in live terrestrial animals \| \| B - National plan for AMR surveillance in place but laboratory and epidemiology capacities for generating, analysing and reporting data are lacking. \| \| C - Some AMR data is collected at local levels but a nationally standardized approach is not used. National coordination and/or quality management is lacking. \| \| D - Priority pathogenic/ commensal bacterial species have been identified for surveillance. Data systematically collected and reported on levels of resistance in at least one of those bacterial species, involving a laboratory that follows quality management processes e.g. proficiency testing. \| \| E - National system of AMR surveillance established for priority animal pathogens, zoonotic and commensal bacterial isolates which follows quality assurance processes in line with intergovernmental standards. Laboratories that report for AMR surveillance follow quality assurance processes. \| |

| **FACTOR CONSISTENCY ASSESSMENT** |
| --- |
| **HIGH**  Minor question change (aquatic systems relatively small component).  Minor changes to response levels.  Some possibility that question wording change may be causing apparent response differences (exclusion of aquatic surveillance). |

## 3 Surveillance Food

| **2019 QUESTION** | **SANKEY – ALL COUNTRIES 2019 - 2024** | **2024 QUESTION (tracked changes)** |
| --- | --- | --- |
| \| ***Question*** \| ***Response options*** \| \| --- \| --- \| \| 7.5 (c) National surveillance system for antimicrobial resistance (AMR) in food (animal and plant origin) \| A - No national plan for a system of surveillance of AMR is available. \| \| B - National plan for surveillance of AMR but capacity (including laboratory and for reporting data on AMR) is lacking. \| \| C - Some AMR data is collected locally but may not use a standardised approach and lacks national coordination and/or quality management. \| \| D - Priority food borne pathogenic/ indicator bacterial species have been identified for surveillance. Data systematically collected and reported on levels of resistance in at least 1 of those bacterial species, involving a laboratory that follows quality management processes, e.g. proficiency testing. \| \| E - National system of surveillance of AMR established for priority foodborne pathogens and/or relevant indicator bacteria which follows quality assurance processes in line with intergovernmental standards. Laboratories that report for AMR surveillance follow quality assurance processes. \| | 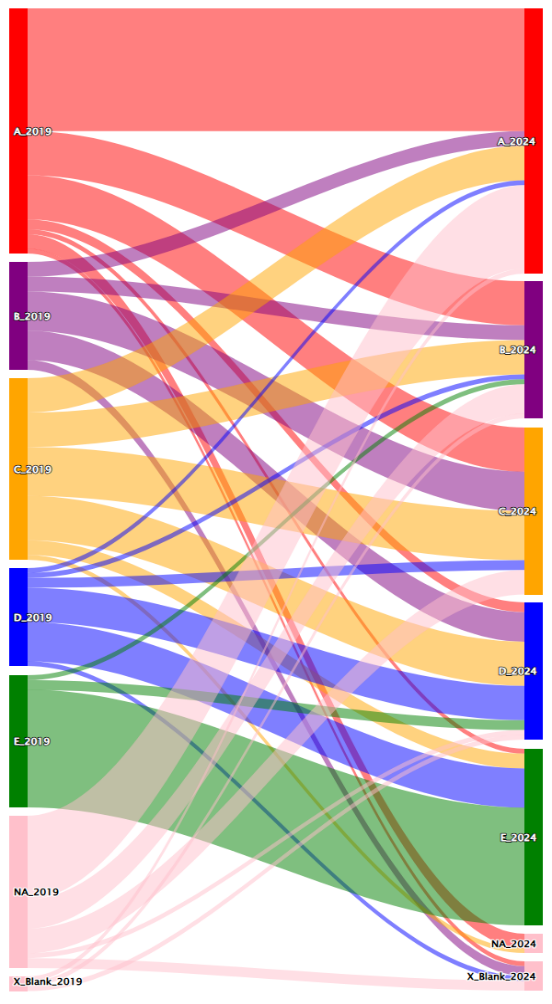 | \| ***Response options*** \| ***Question*** \| \| --- \| --- \| \| A - No national plan for an AMR surveillance system. \| 5.3 National surveillance system for antimicrobial resistance (AMR) in food (terrestrial and aquatic animal and plant origin) \| \| B - National plan for AMR surveillance in place but capacity (including laboratory and reporting) is lacking. \| \| C - Some AMR data is collected - but a standardized approach is not used. National coordination and/or quality management is lacking. \| \| D - Priority food borne pathogenic/ indicator bacterial species have been identified for surveillance. Data systematically collected and reported on levels of resistance in at least one of those bacterial species, involving a laboratory that follows quality management processes e.g. proficiency testing. \| \| E - National system of AMR surveillance established for priority foodborne pathogens and/or relevant indicator bacteria which follows quality assurance processes in line with intergovernmental standards. Laboratories that report for AMR surveillance follow quality assurance processes. \| |

| **FACTOR CONSISTENCY ASSESSMENT** |
| --- |
| **FULL**  Question effectively identical.  Response levels effectively identical.  No reason to consider that factor changes may cause apparent response differences. |

## 4 OH collaboration

| **2019 QUESTION** | **SANKEY – ALL COUNTRIES 2019 - 2024** | **2024 QUESTION (tracked changes)** |
| --- | --- | --- |
| \| ***Question*** \| ***Response options*** \| \| --- \| --- \| \| 4.1 Multi-sector and One Health collaboration/coordination \| A - No formal multi-sectoral governance or coordination mechanism on AMR exists. \| \| B - Multi-sectoral working group(s) or coordination committee on AMR established with Government leadership. \| \| C - Multi-sectoral working group(s) is (are) functional, with clear terms of reference; regular meetings, and funding for working group(s). Activities and reporting/accountability arrangements are defined. \| \| D - Joint working on issues including agreement on common objectives. \| \| E - Integrated approaches used to implement the national AMR action plan with relevant data and lessons learned from all sectors used to adapt implementation of the action plan. \| | 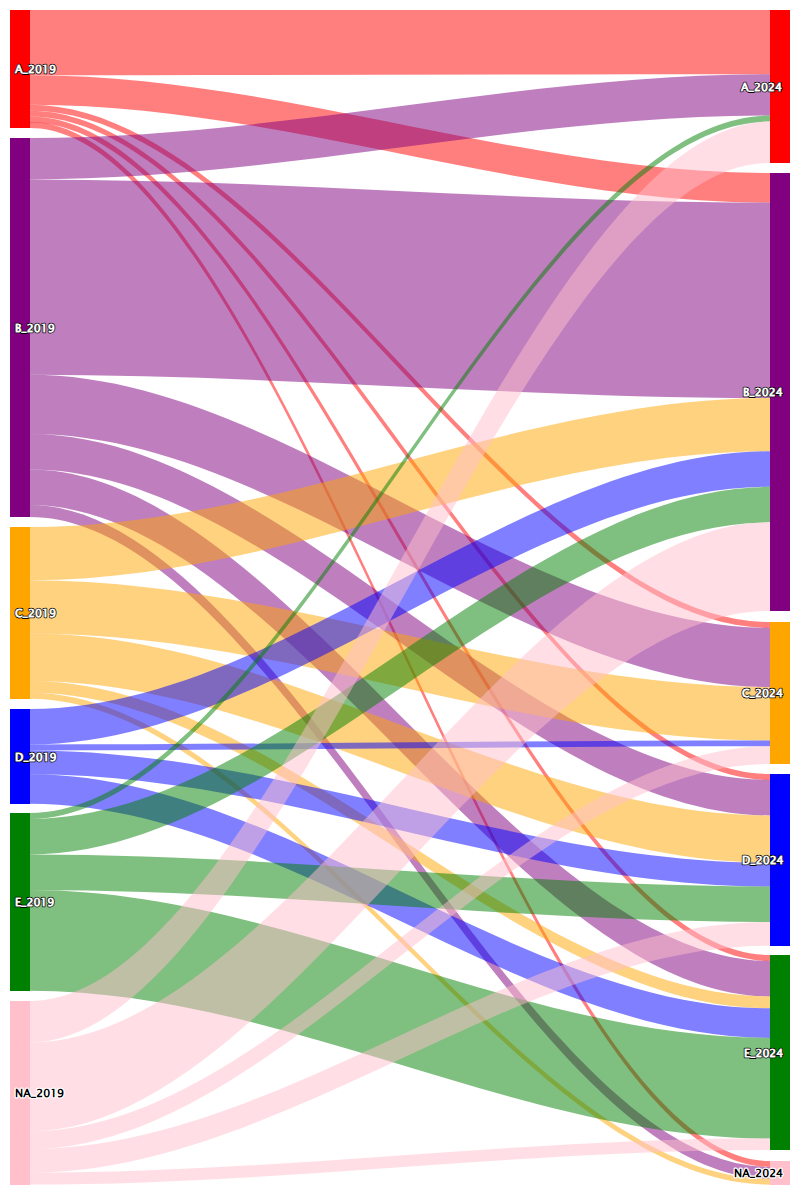 | \| ***Response options*** \| ***Question*** \| \| --- \| --- \| \| A - No formal multi-sectoral governance or coordination mechanism on AMR exists. \| 2.1Multi-sector and One Health collaboration/coordination \| \| B - Multi-sectoral coordination mechanism on AMR established with Government leadership. \| \| C - Formalized Multisector coordination mechanism with technical working groups established with clear terms of reference, regular meetings, and funding for working group(s) with activities and reporting/accountability arrangements defined. \| \| D - Joint working on issues including agreement on common objectives. \| \| E - Integrated approaches used to implement the national AMR action plan with relevant data and lessons learned from all sectors used to adapt implementation of the action plan. \| |

| **FACTOR CONSISTENCY ASSESSMENT** |
| --- |
| **HIGH**  Identical question.  Minor changes to response levels.  Some possibility that response level changes may be causing apparent response differences (option C more complicated). |

## 5 NAP progress

| **2019 QUESTION** | **SANKEY – ALL COUNTRIES 2019 - 2024** | **2024 QUESTION (tracked changes)** |
| --- | --- | --- |
| \| ***Question*** \| ***Response options*** \| \| --- \| --- \| \| 5.1 Country progress with development of a national action plan on AMR \| A - No national AMR action plan. \| \| B - National AMR action plan under development. \| \| C - National AMR action plan developed. \| \| D - National AMR action plan approved by government that reflects Global Action Plan objectives, with an operational plan and monitoring arrangements. \| \| E - National AMR action plan has funding sources identified, is being implemented and has relevant sectors involved with a defined monitoring and evaluation process in place. \| | 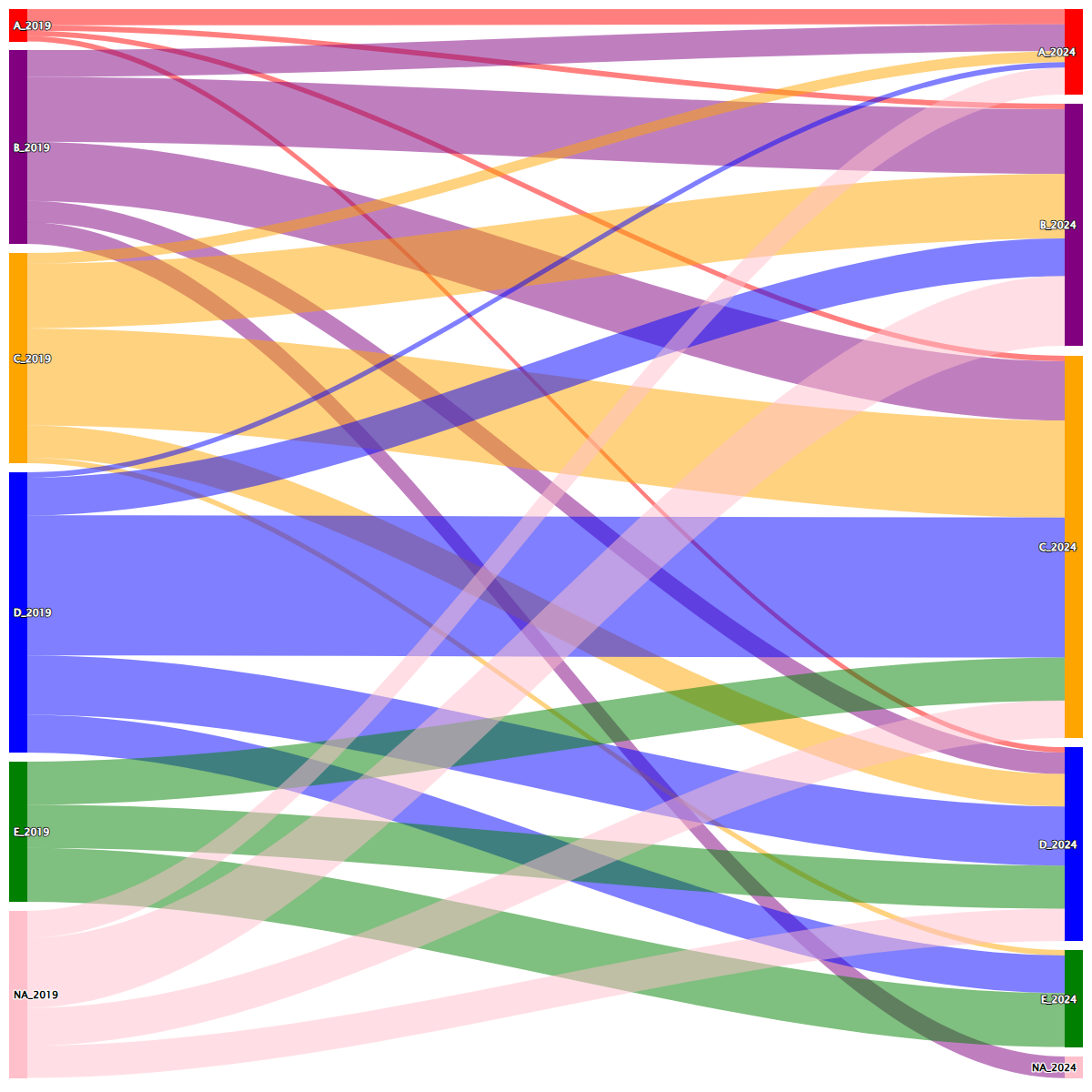 | \| ***Response options*** \| ***Question*** \| \| --- \| --- \| \| A - No national AMR action plan or plan under development. \| 2.3 Country progress with development of a national action plan on AMR \| \| B - National AMR action plan developed. \| \| C - National AMR action plan approved by government and is being implemented. \| \| D - National AMR action plan has costed and budgeted operational plan and has monitoring mechanism in place. \| \| E - Financial provision for the National AMR action plan implementation is included in the national plans and budgets. \| |

| **FACTOR CONSISTENCY ASSESSMENT** |
| --- |
| **MODERATE**  Identical question.  Significant changes to response levels: ‘under development’ moved from B to A; approval and implementation required for C; more specific financial requirements in D and E.  Plausible that response level changes may be causing apparent response differences (2024 scale more demanding). |

## 6 NAP integration

| **2019 QUESTION** | **SANKEY – ALL COUNTRIES 2019 - 2024** | **2024 QUESTION (tracked changes for question not responses)** |
| --- | --- | --- |
| \| ***Question*** \| ***Response options*** \| \| --- \| --- \| \| 5.2 Is your country’s national action plan on AMR linked to any other existing action plans, strategies or targets related to HIV, tuberculosis, malaria or neglected tropical diseases? If so, please select the relevant item (mark all diseases that are relevant): \| [HIV] \| \| [Tuberculosis] \| \| [Malaria ] \| \| [Neglected tropical diseases] \| | 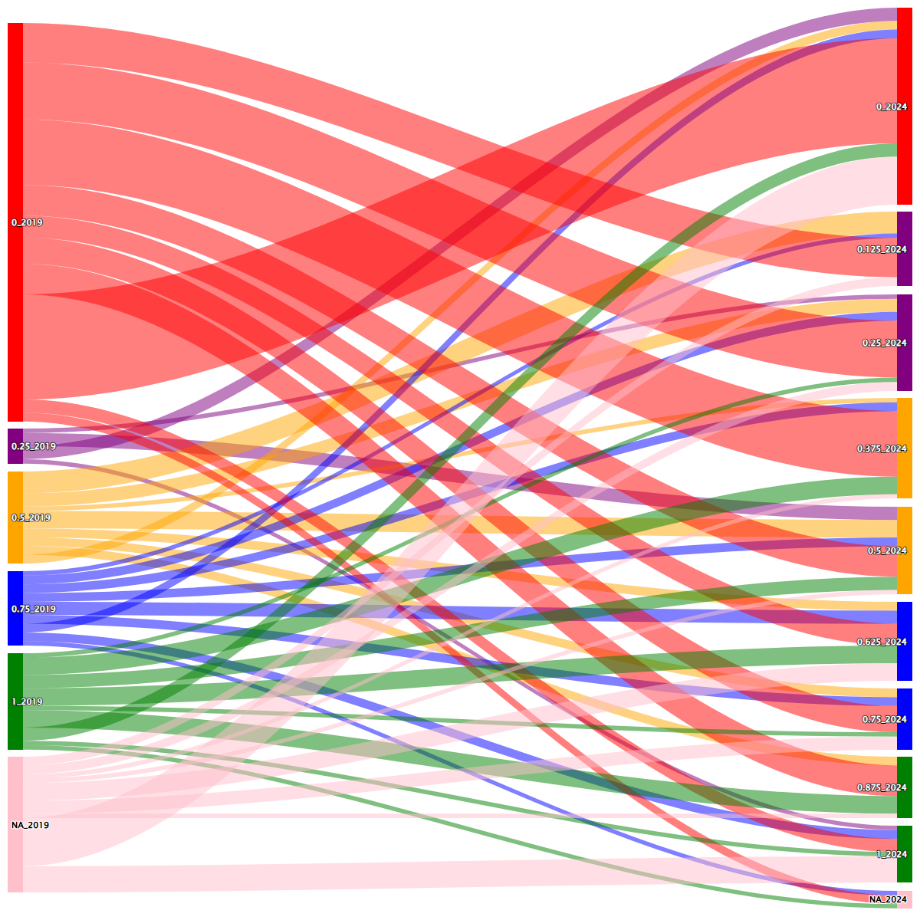 | \| ***Response options*** \| ***Question*** \| \| --- \| --- \| \| [One Health Strategy or One Health mechanism] \| 2.6 Is your country’s national planning on AMR integrated with other existing action plans or, strategies?  …  NB not including: [Other]  [If other, please specify] \| \| [Water, Sanitation and Hygiene (WASH)] \| \| [National health sector plan] \| \| [National action plan on health security] \| \| [Climate change and environmental planning] \| \| [National development plans; United Nations Sustainable Development Coorporation Framework (UNSDCF)] \| \| [National Food Safety strategy and policies] \| \| [National Agriculture development plans and policies] \| |

| **FACTOR CONSISTENCY ASSESSMENT** |
| --- |
| **LOW**  Similar question subject, but wording and structure completely revised.  Completely different response levels.  Apparent response differences give only approximate sense of degree to which country AMR plans associated with other plans considered by WHO to be relevant in given year. |

## 7 Raising awareness

| **2019 QUESTION** | **SANKEY – ALL COUNTRIES 2019 - 2024** | **2024 QUESTION (tracked changes)** |
| --- | --- | --- |
| \| ***Question*** \| ***Response options*** \| \| --- \| --- \| \| 6.1 Raising awareness and understanding of AMR risks and response \| A - No significant awareness-raising activities on relevant aspects of risks of antimicrobial resistance. \| \| B - Some activities in parts of the country to raise awareness about risks of antimicrobial resistance and actions that can be taken to address it. \| \| C - Limited or small-scale antimicrobial resistance awareness campaign targeting some but not all relevant stakeholders. \| \| D - Nationwide, government-supported antimicrobial resistance awareness campaign targeting all or the majority of relevant stakeholders, based on stakeholder analysis, utilizing targeted messaging accordingly within sectors. \| \| E - Targeted, nationwide government-supported activities implemented to change behavior of key stakeholders within sectors, with monitoring undertaken over the last 2-5 years. \| | 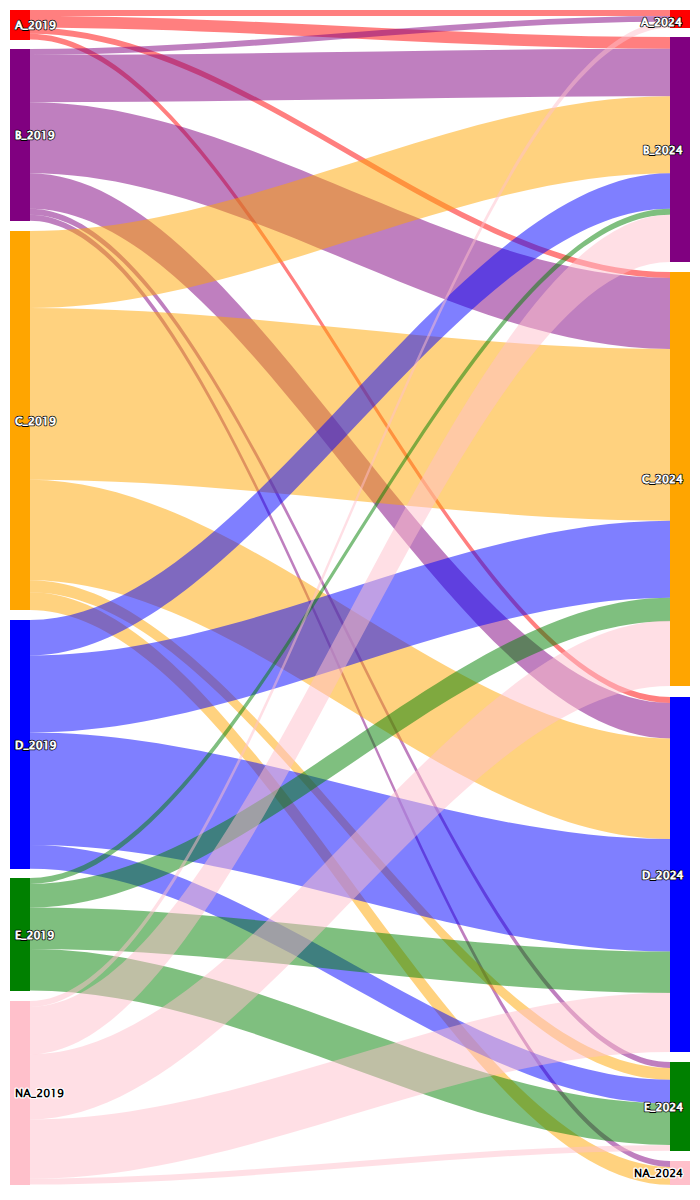 | \| ***Response options*** \| ***Question*** \| \| --- \| --- \| \| A - No awareness-raising activities on risks of antimicrobial resistance. \| 2.9 Raising awareness and understanding of AMR risks and response \| \| B - Some activities to raise awareness about risks of antimicrobial resistance and actions that address it. \| \| C - Some awareness activities at local and/or sub-national level about risks of antimicrobial resistance and actions to address it, targeting some but not all relevant stakeholders, based on stakeholder analysis. \| \| D - Nationwide, government-supported antimicrobial resistance awareness raising campaign targeting all or the majority of priority stakeholder groups, utilizing targeted messaging accordingly within sectors. \| \| E - Routine Targeted, nationwide government-supported campaign implemented to raise awareness of priority stakeholders across sectors, with regular monitoring. \| |

| **FACTOR CONSISTENCY ASSESSMENT** |
| --- |
| **FULL**  Identical question.  Minor changes to response levels.  No reason to consider that response level changes may cause apparent response differences. |

## 8 Data decisions HH

| **2019 QUESTION** | **SANKEY – ALL COUNTRIES 2019 - 2024** | **2024 QUESTION** |
| --- | --- | --- |
| \| ***Question*** \| ***Response options*** \| ***Numeric value*** \| \| --- \| --- \| --- \| \| 7.6 Multi-sectoral working group or coordination committee in charge of national AMR strategy reviews data on antimicrobial consumption and resistance in human and animal sectors at least annually, considers implications for and amends national strategy accordingly [For human health] \| No \| 0 \| \| Yes \| 1 \| | 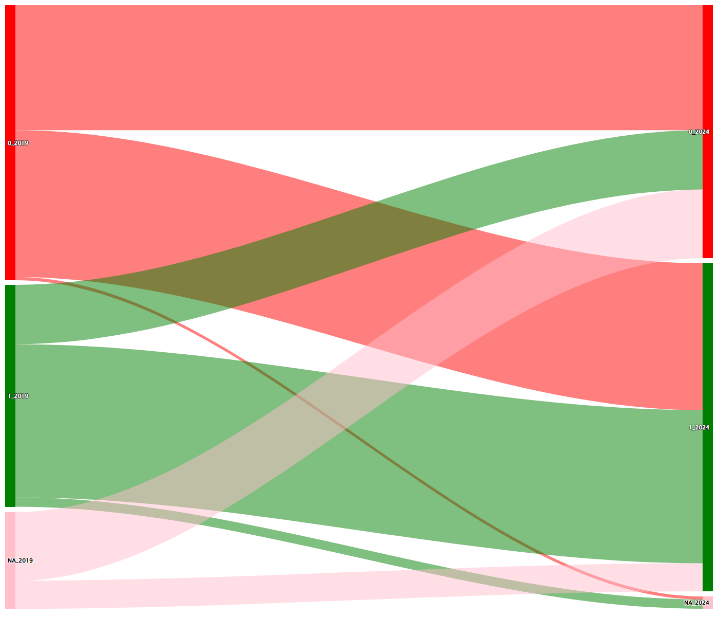 | \| ***Numeric value*** \| ***Response options*** \| ***Question*** \| \| --- \| --- \| --- \| \| 0 \| No, No  No, Yes  Yes, No \| 2.11 Is the country using relevant antimicrobial consumption/use data…  **AND**  2.12 Is the country using relevant antimicrobial resistance surveillance data…  …. to inform operational decision making and amend policies  "If yes, for which sector/s"  "Human Health" \| \| 1 \| Yes AND Yes \| |

| **FACTOR CONSISTENCY ASSESSMENT** |
| --- |
| **LOW**  Similar question subject, but wording and structure completely revised.  Response levels are Yes/No in both cases, but 2024 response includes responses to two questions.  Likely that factor changes cause apparent response differences (2024 questions only require ‘use’ of data, more stringent conditions in 2019; but 2024 question processing also requires both AMC/U and AMR data to be used, less specific on exact nature of data in 2019) |

## 9 Data decisions AH

| **2019 QUESTION** | **SANKEY – ALL COUNTRIES 2019 - 2024** | **2024 QUESTION** |
| --- | --- | --- |
| \| ***Question*** \| ***Response options*** \| ***Numeric value*** \| \| --- \| --- \| --- \| \| 7.6 Multi-sectoral working group or coordination committee in charge of national AMR strategy reviews data on antimicrobial consumption and resistance in human and animal sectors at least annually, considers implications for and amends national strategy accordingly [For animal health] \| No \| 0 \| \| Yes \| 1 \| | 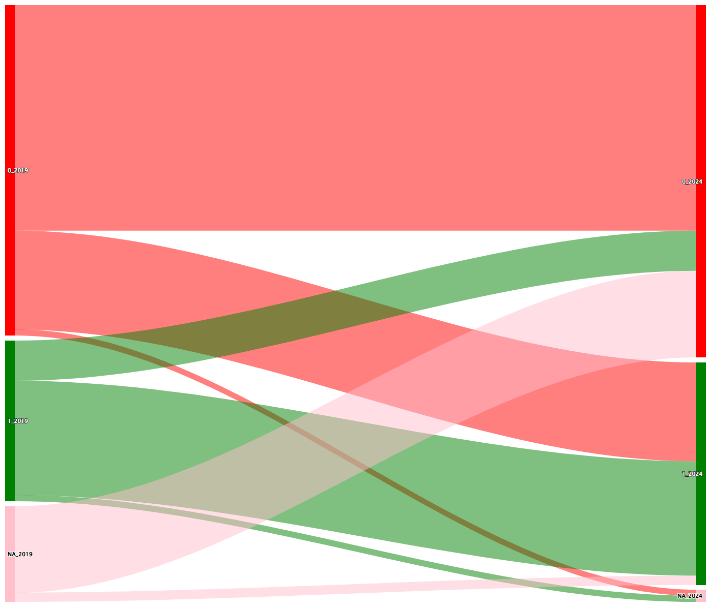 | \| ***Numeric value*** \| ***Response options*** \| ***Question*** \| \| --- \| --- \| --- \| \| 0 \| No, No  No, Yes  Yes, No \| 2.11 Is the country using relevant antimicrobial consumption/use data…  **AND**  2.12 Is the country using relevant antimicrobial resistance surveillance data…  …. to inform operational decision making and amend policies  "If yes, for which sector/s"  "Animal Health" \| \| 1 \| Yes AND Yes \| |

| **FACTOR CONSISTENCY ASSESSMENT** |
| --- |
| **LOW**  See Topic 8: same assessment. |

## 10 IPC programme HH

| **2019 QUESTION** | **SANKEY – ALL COUNTRIES 2019 - 2024** | **2024 QUESTION (no changes)** |
| --- | --- | --- |
| \| ***Question*** \| ***Response options*** \| \| --- \| --- \| \| 8.1 Infection Prevention and Control (IPC) in human health care \| A - No national IPC programme or operational plan is available. \| \| B - A national IPC programme or operational plan is available. National IPC and water, sanitation and hygiene (WASH) and environmental health standards exist but are not fully implemented. \| \| C - A national IPC programme and operational plan are available and national guidelines for health care IPC are available and disseminated. Selected health facilities are implementing the guidelines, with monitoring and feedback in place. \| \| D - National IPC programme available according to the WHO IPC core components guidelines+ and IPC plans and guidelines implemented nationwide. All health care facilities have a functional built environment (including water and sanitation), and necessary materials and equipment to perform IPC, per national standards. \| \| E - IPC programmes are in place and functioning at national and health facility levels according to the WHO IPC core components guidelines. Compliance and effectiveness are regularly evaluated and published. Plans and guidance are updated in response to monitoring. \| | 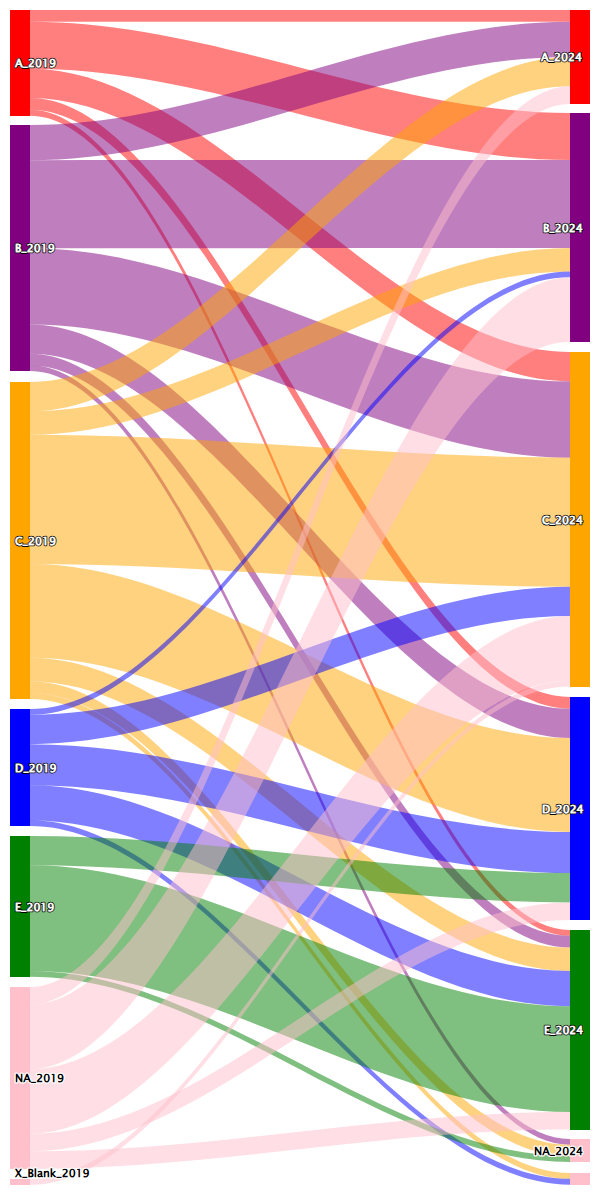 | \| ***Response options*** \| ***Question*** \| \| --- \| --- \| \| A - No national IPC programme or operational plan is available. \| 3.5 Infection Prevention and Control (IPC) in human health care \| \| B - A national IPC programme or operational plan is available. National IPC and water, sanitation and hygiene (WASH) and environmental health standards exist but are not fully implemented. \| \| C - A national IPC programme and operational plan are available and national guidelines for health care IPC are available and disseminated. Selected health facilities are implementing the guidelines, with monitoring and feedback in place. \| \| D - National IPC programme available according to the WHO IPC core components guidelines* and IPC plans and guidelines implemented nationwide. All health care facilities have a functional built environment (including water and sanitation), and necessary materials and equipment to perform IPC, per national standards. \| \| E - IPC programmes are in place and functioning at national and health facility levels according to the WHO IPC core components guidelines. Compliance and effectiveness are regularly evaluated and published. Plans and guidance are updated in response to monitoring. \| |

| **FACTOR CONSISTENCY ASSESSMENT** |
| --- |
| **FULL**  Identical question.  Response levels identical.  No reason to consider that factor changes may cause apparent response differences. |

## 11 AMS programme HH

| **2019 QUESTION** | **SANKEY – ALL COUNTRIES 2019 - 2024** | **2024 QUESTION (tracked changes)** |
| --- | --- | --- |
| \| ***Question*** \| ***Response options*** \| \| --- \| --- \| \| 9.1 Optimizing antimicrobial use in human health \| A - No/weak national policies for appropriate use. \| \| B - National policies for antimicrobial governance developed for the community and health care settings. \| \| C - Practices to assure appropriate antimicrobial use being implemented in some healthcare facilities and guidelines for appropriate use of antimicrobials available. \| \| D - Guidelines and other practices to enable appropriate use are implemented in most health facilities nationwide. Monitoring and surveillance results are used to inform action and to update treatment guidelines and essential medicines lists. \| \| E - Guidelines on optimizing antibiotic use are implemented for all major syndromes and data on use is systematically fed back to prescribers. \| | 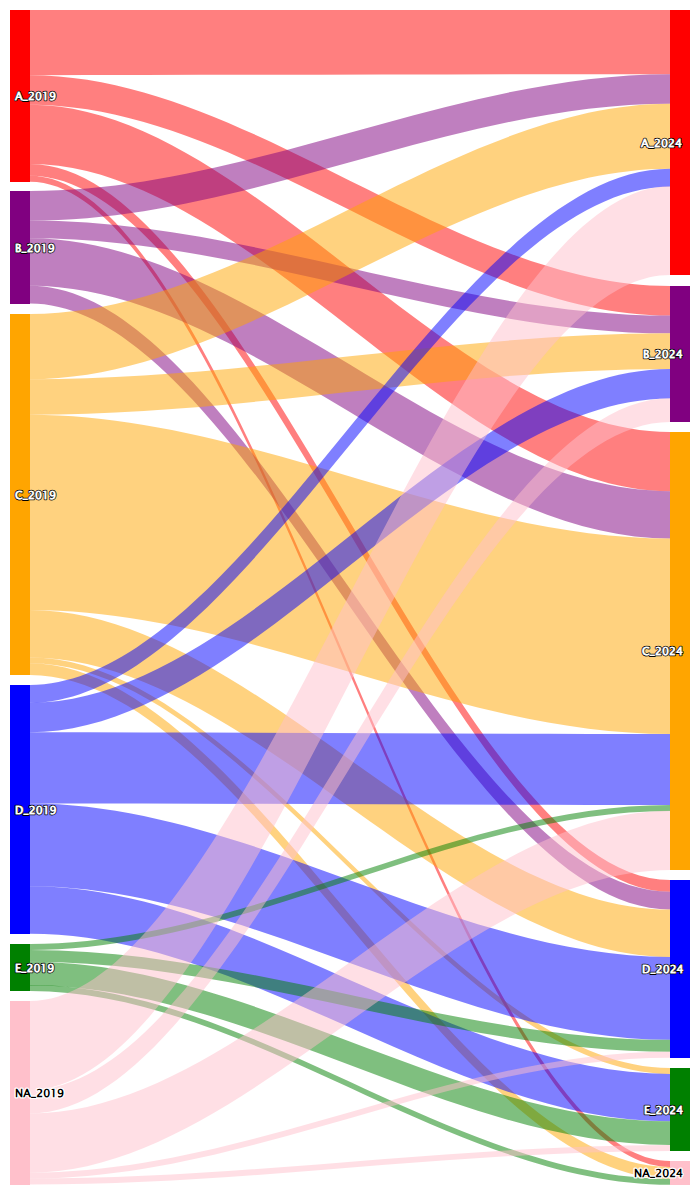 | \| ***Response options*** \| ***Question*** \| \| --- \| --- \| \| A - No/weak national policies† for appropriate antimicrobial use including availability, quality, and disposal of antimicrobials. \| 3.6 Optimizing antimicrobial use in human health \| \| B - National policies† promoting appropriate antimicrobial use/antimicrobial stewardship activities developed for the community and health care settings. \| \| C - National guidelines for appropriate use of antimicrobials are available and antimicrobial stewardship programs are being implemented in some healthcare facilities. \| \| D - National guidelines for appropriate use of antimicrobials are available and antimicrobial stewardship programs are being implemented in most health care facilities nationwide. Monitoring and surveillance results§ are used to inform action and to update treatment guidelines and essential medicines lists. \| \| E - National guidelines on optimizing antibiotic use are implemented for all major syndromes and data on use is systematically fed back to prescribers. \| |

| **FACTOR CONSISTENCY ASSESSMENT** |
| --- |
| **HIGH**  Identical question.  Minor changes to response levels.  Some possibility that response level changes may be causing apparent response differences (specific examples added to level A of areas in which policy might be absent or weak.). |

## 12 Practices AH

| **2019 QUESTION** | **SANKEY – ALL COUNTRIES 2019 - 2024** | **2024 QUESTION (tracked changes)** |
| --- | --- | --- |
| \| ***Question*** \| ***Response options*** \| \| --- \| --- \| \| 8.2 Good health, management and hygiene practices to reduce the use of antimicrobials and minimize development and transmission of AMR in animal production (terrestrial and aquatic) \| A - No systematic efforts to improve good production practices. \| \| B - Some activities in place to develop and promote good production practices. \| \| C - National plan agreed to ensure good production practices in line with international standards (e.g. OIE Terrestrial and Aquatic Codes, Codex Alimentarius). Nationally agreed guidance for good production practices developed, adapted for implementation at local farm and food production level. \| \| D - Nationwide implementation of plan to ensure good production practices and national guidance published and disseminated. \| \| E - Nationwide implementation of plan to ensure good production practices and monitoring of impact on level of AMR, on animal health and welfare, and on production, with updating of plans and guidance in response to findings. \| | 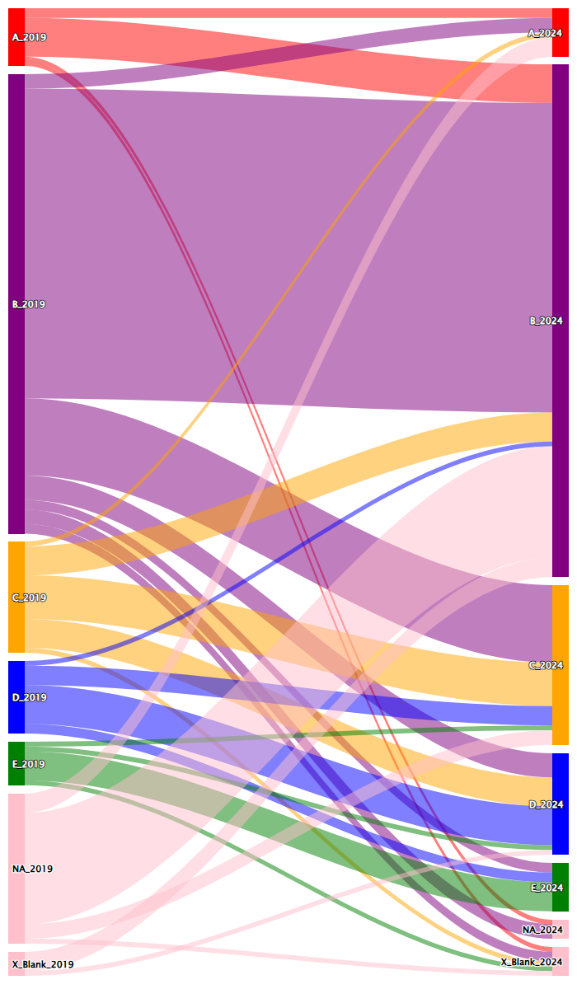 | \| ***Response options*** \| ***Question*** \| \| --- \| --- \| \| A - No systematic efforts to improve good animal husbandry and biosecurity practices. \| 4.9 Biosecurity† and good animal husbandry practices‡ to reduce the use of antimicrobials and minimize development and transmission of AMR in terrestrial animal production \| \| B - Some activities in place to develop and promote good animal husbandry and biosecurity practices. \| \| C - National plan agreed to ensure good animal husbandry and biosecurity practices in line with international standards (e.g. WOAH Terrestrial/Aquatic Codes, Codex Alimentarius). Nationally agreed guidance for good practices developed, adapted for implementation at local farm and food production level. \| \| D - Nationwide implementation of plan to ensure good animal husbandry and biosecurity practices and national guidance published and disseminated. \| \| E - Implementation of the nation-wide plan is monitored periodically. \| |

| **FACTOR CONSISTENCY ASSESSMENT** |
| --- |
| **MODERATE**  Similar question subject, but wording changed (from ‘good health, management and hygiene practices’ to ‘biosecurity and good animal husbandry practices’).  Question wording change implemented at all levels of response options (no longer addressing ‘production’ generally).  Plausible that factor changes may be causing apparent response differences (2024 question & response levels require more specific change). |

## 13 Regulations HH

| **2019 QUESTION** | **SANKEY – ALL COUNTRIES 2019 - 2024** | **2024 QUESTION (tracked changes)** |
| --- | --- | --- |
| \| ***Question*** \| ***Response options*** \| \| --- \| --- \| \| 5.4 Country has laws or regulations on prescription and sale of antimicrobials, for human use \| No \| \| Yes \| | 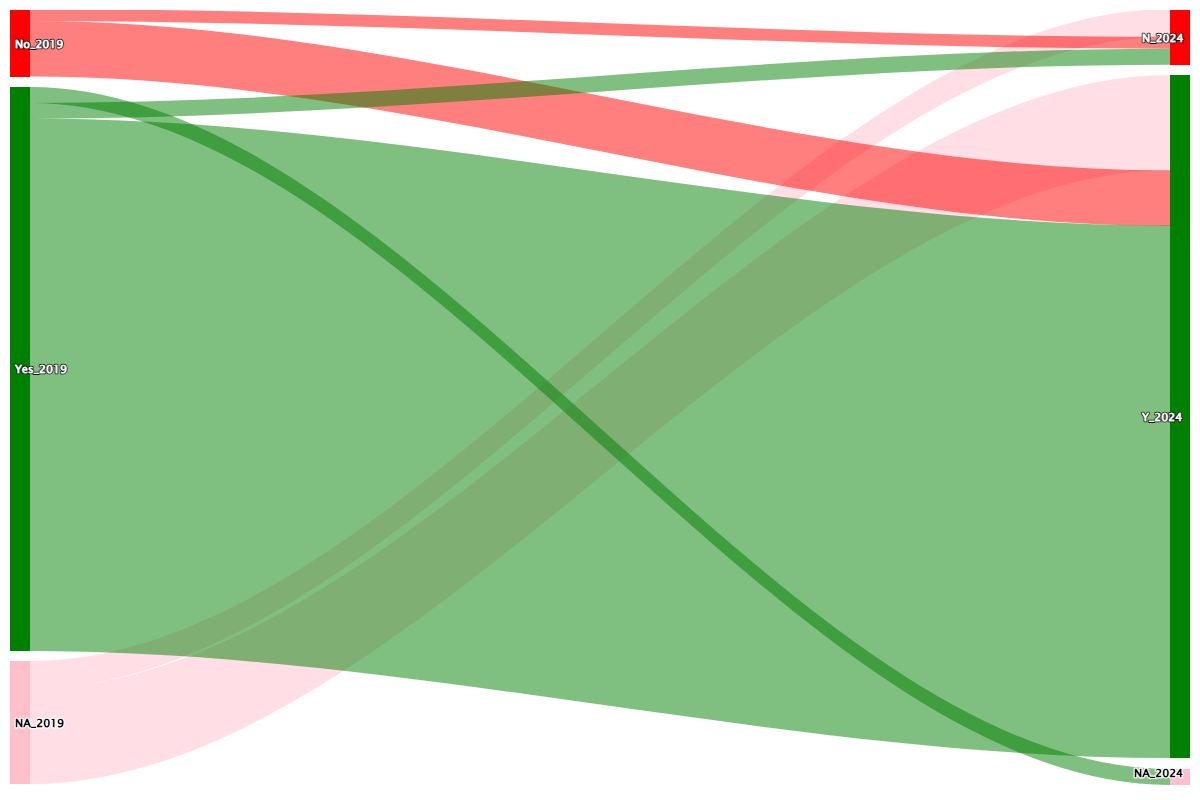 | \| ***Response options*** \| ***Question*** \| \| --- \| --- \| \| No \| 2.8.1 Country has laws or regulations on prescription and sale of antimicrobials, for human use \| \| Yes \| |

| **FACTOR CONSISTENCY ASSESSMENT** |
| --- |
| **FULL**  Identical question.  Response levels identical.  No reason to consider that factor changes may cause apparent response differences. |

## 14 Regulations AH

| **2019 QUESTION** | **SANKEY – ALL COUNTRIES 2019 - 2024** | **2024 QUESTION** |
| --- | --- | --- |
| \| ***Question*** \| ***Response options*** \| ***Numeric value*** \| \| --- \| --- \| --- \| \| 5.4 Country has laws or regulations on prescription and sale of antimicrobials for animal use. \| No \| 0 \| \| Yes \| 1 \| | 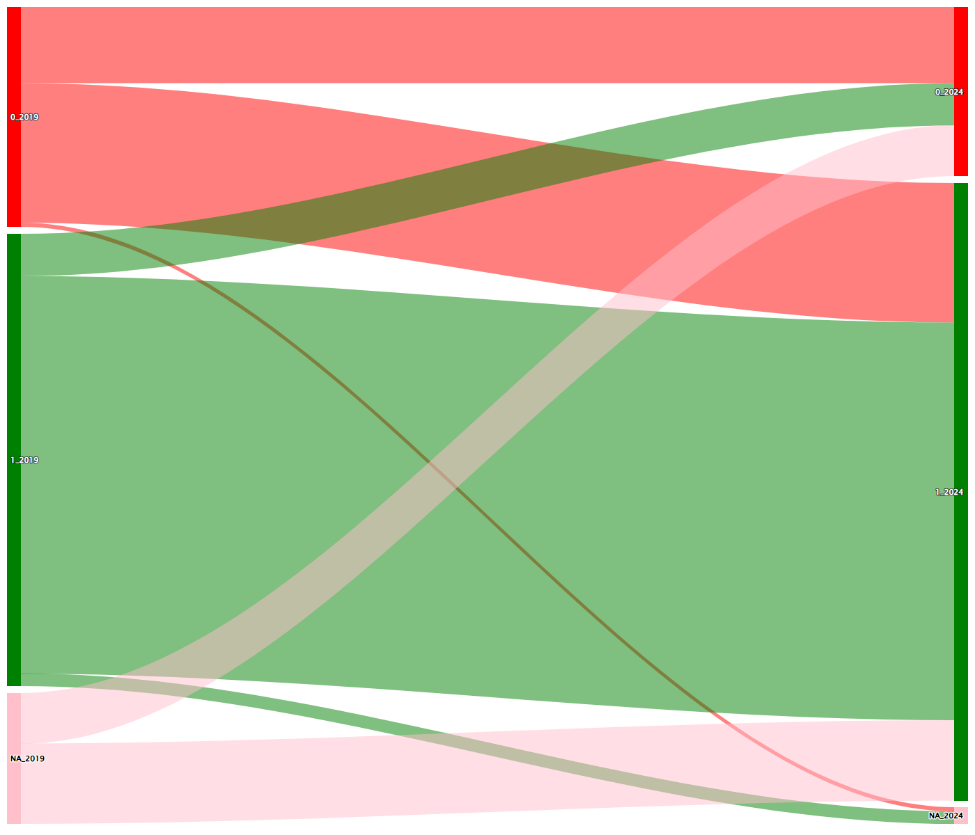 | \| ***Numeric value*** \| ***Response options*** \| ***Question*** \| \| --- \| --- \| --- \| \| 0 \| both = No \| "2.8.2 Country has laws or regulations on prescription and sale of antimicrobials for terrestrial animal use.  **OR**  2.8.3 Country has laws or regulations on prescription and sale of antimicrobials for aquatic animals." \| \| 1 \| Yes OR Yes \| |

| **FACTOR CONSISTENCY ASSESSMENT** |
| --- |
| **HIGH**  Minor question wording change.  Response levels are Yes/No in both cases, but 2024 response includes responses to two questions.  Possible that factor changes cause apparent response differences (2019 responses about animal use might in theory have reflected laws or regulations for non-terrestrial or aquatic) |

## 15 Regulations AH (growth)

| **2019 QUESTION** | **SANKEY – ALL COUNTRIES 2019 - 2024** | **2024 QUESTION (tracked changes)** |
| --- | --- | --- |
| \| ***Question*** \| ***Response options*** \| \| --- \| --- \| \| 5.4 Country has laws or regulations that prohibits the use of antibiotics for growth promotion in the absence of risk analysis. \| No \| \| Yes \| | 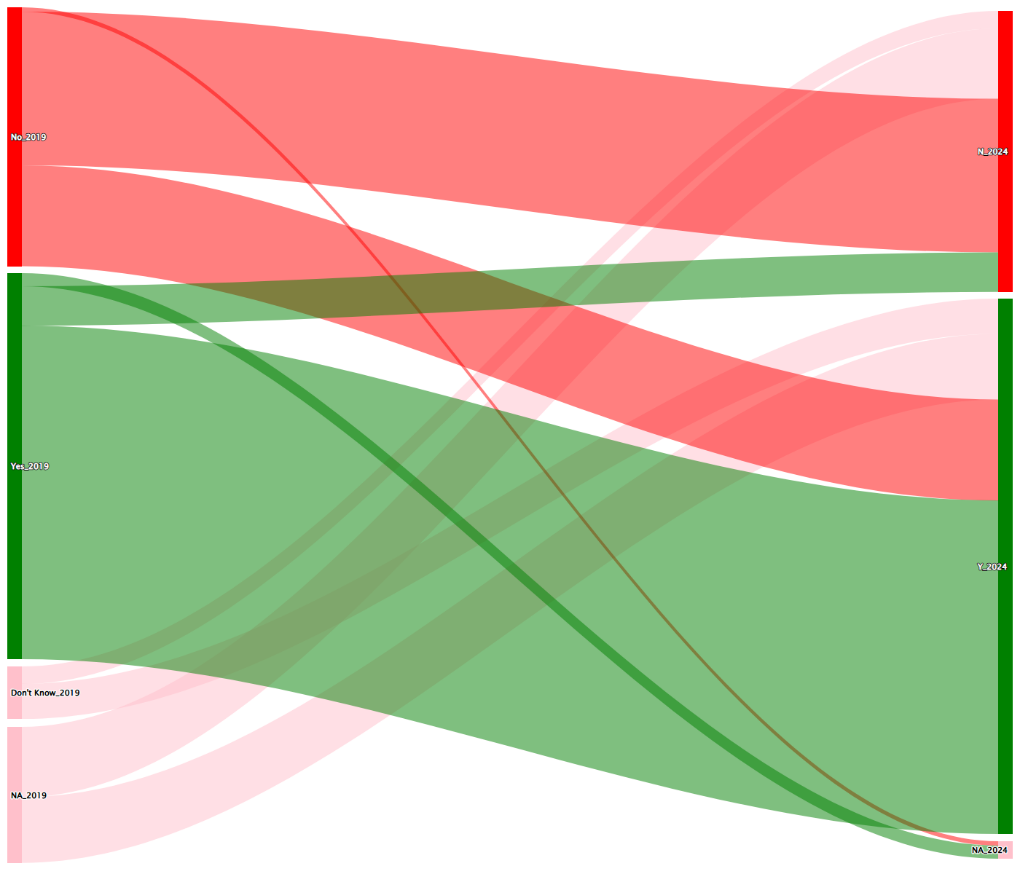 | \| ***Response options*** \| ***Question*** \| \| --- \| --- \| \| No \| 2.8.5 Country has laws or regulations that prohibits the use of antibiotics for growth promotion in terrestrial animals in the absence of risk analysis. \| \| Yes \| |

| **FACTOR CONSISTENCY ASSESSMENT** |
| --- |
| **HIGH**  Question very similar (added focus on terrestrial animals).  Response levels identical.  Possible that question change causes apparent response differences (although unclear whether 2024 standard stricter or not) |
